# Interrogating the validity of cumulative indices of environmental and genetic risk for negative developmental outcomes

**DOI:** 10.1017/S0954579421001097

**Published:** 2021-12-13

**Authors:** Keith F. Widaman

**Affiliations:** University of California, Riverside, CA 92521, USA

**Keywords:** environmental risk, G×E interaction, genetic risk, regression analysis, risk indices

## Abstract

Indices of cumulative risk (CR) have long been used in developmental research to encode the number of risk factors a child or adolescent experiences that may impede optimal developmental outcomes. Initial contributions concentrated on indices of cumulative environmental risk; more recently, indices of cumulative genetic risk have been employed. In this article, regression analytic methods are proposed for interrogating strongly the validity of risk indices by testing optimality of compositing weights, enabling more informative modeling of effects of CR indices. Reanalyses of data from two studies are reported. One study involved 10 environmental risk factors predicting Verbal IQ in 215 four-year-old children. The second study included an index of genetic CR in a G×E interaction investigation of 281 target participants assessed at age 15 years and then again at age 31 years for observed hostility during videotaped interactions with close family relations. Principles to guide evaluation of results of statistical modeling are presented, and implications of results for research and theory are discussed. The ultimate goals of this paper are to develop stronger tests of conjectures involving CR indices and to promote methods for improving replicability of results across studies.

The notion of risks to optimal developmental outcomes has been a topic of considerable interest in the field of developmental psychopathology for over half a century, if not longer. Head Start programs were begun in the 1960s in an attempt to remediate environmental shortcomings for children from disadvantaged backgrounds, under the assumption that various environmental factors that presage poorer educational outcomes might be mitigated through the development of early childhood programs of instruction and enrichment (LOVE, CHAZAN-COHEN, & RAIKES, 2007; BEATTY & ZIGLER, 2012). Several years earlier, the Collaborative Perinatal Project (CPP) was initiated in 1958 under the assumption that a *continuum of reproductive casualty* may account for many poor birth and early child outcomes, and over 50,000 women and their offspring were eventually recruited into the CPP (BROMAN, NICHOLS, & KENNEDY, 1975; BROMAN, 1987). The primary focus of the CPP was risk factors for outcomes such as cerebral palsy, intellectual disability, and low general intellectual functioning. The host of risk factors invoked in reproductive casualty involved many presumptive harmful factors spanning the prenatal (e.g., poor prenatal nutrition), perinatal (e.g., anoxia, improper use of forceps), and postnatal periods (e.g., poor infant nutrition, low SES).

Extending the scope of risk factors, Sameroff and Chandler (1975) coined the term *continuum of caretaking casualty*, hypothesizing that a large number of environmental risk factors are hazards to optimal child development. The theoretical model developed by Sameroff and Chandler involved transactions among the child, the parents, and the environment within which the family was living. Sameroff and Chandler emphasized that, although many reproductive risks certainly should be considered, no adequate accounting of intellectual or other developmental deficits has stemmed directly only from reproductive risks. In the vast majority of cases, however, an array of caretaking risks appear to be required for an adequate representation of the course of impaired development during infancy, childhood, and adolescence. These caretaking risk factors characterize the environment within which the child is developing, leading to indexes often identified as indices of environmental cumulative risk (CR).

More recently, research on gene and gene X environment (G×E) interaction effects on behavior has increased exponentially as a large number of single nucleotide polymorphisms (SNPs) can be obtained from the human genome. Each SNP can be scored in a discrete fashion, indicating the number of particular alleles present for that SNP. Many studies utilizing SNPs as the basis for G×E testing have conducted analyses on only one or another of select target SNPs. However, building on successful use of environmental risk indices, some researchers have promoted the use of summative scores across multiple SNPs, which yield scores on an index of genetic CR (e.g., Belsky & Beaver, 2011). More complicated analyses use results from genome-wide association studies (GWASs) to supply weights when forming a genome-wide index of genetic CR (Belsky & Harden, 2019).

The idea of formulating a risk index was based on observations by clinically oriented researchers. For example, Rutter (1981) noted that children seem relatively resilient to experiencing one or a small number of risk factors. But, as the number of risk factors to which a child is exposed increases, the likelihood of negative developmental outcomes increases. In one of the earliest studies using a risk index for caretaking casualty, Sameroff, Seifer, Barocas, Zax, and Greenspan (1987) identified 10 risk factors for low intelligence, scored each risk factor in dichotomous fashion (0 = low risk, 1 = high risk), and summed the risk factors into a cumulative index that could range from 0 to 10, representing the number of risks a child faced. They then contrasted alternate regression models, finding that the use of all 10 risk factors allowed stronger prediction of 4-year-old children’s Verbal IQ than any single predictor. Given the success of the Sameroff et al. research, the use of risk indices has burgeoned over the past three decades.

A key issue in use of a CR index is the optimal weighting of the components of the index. CR indices have been constructed in many ways across studies. Evans, Li, and Whipple (2013) provided a comprehensive review of theory, methodological concerns, and research findings using CR indices. The current paper builds on the work of Sameroff et al. (1987), Evans et al. (2013), and others to consider in more detail several issues of importance in the construction and evaluation of CR indices. Specifically, statistical methods are described for interrogating, or testing severely, the validity of CR indices as typically formed. Mayo (2018) recently advocated severe testing of theoretical conjectures, arguing that severe testing can uncover problems that deserve attention, problems that might be masked with less severe testing. Furthermore, if conjectures are tested severely and survive these tests, firmer inductive support for the conjectures accrues. In a related tone, Rodgers (2019) argued that data might profitably be conceptualized as valuable capital and that an investigator spends some capital when estimating each parameter in a statistical model. In effect, one invests capital in the process of estimating each parameter, and a researcher should be vigilant to assess the return on investment in terms of the quality of the resulting model, including its estimates and their associated *SE*s.

The current manuscript first presents issues involved in forming a CR risk from multiple indicators, beginning with the scaling of indicators of risk. Then, methods are described for testing severely or interrogating a risk index, which involves crucial tests of how CR index composites are optimally formed. Following this, two empirical examples are described, one interrogating an index of environmental CR, and the second an index of genetic CR. Discussion revolves around recommendations for stronger testing of theoretical conjectures. If CR indices are tested more severely and informatively and pass these tests, this may also promote successful replication efforts across studies, addressing current pressing questions about the replicability of research in many areas of psychology.

## Forming an index of cumulative risk

Indices of CR are formed from multiple indicators, which can be derived in multiple forms and from multiple domains. All aspects of the formation of CR indices and their use in analytic models should be scrutinized to ensure that researchers benefit most from their use.

### Discrete versus continuous indicators

One of the first matters confronting researchers using CR indicators is the scaling of the indicators included in the index. In perhaps the first study to use a CR index, Sameroff et al. (1987) employed discrete indicators, each scored in dichotomous fashion. Later researchers have attempted to use indicators scaled in more continuous or quantitative fashion. Each method has strengths, and each has weaknesses, all worthy of critical appraisal.

### Discrete or categorical indicators

If Shakespeare had been a practicing scientist, he might have observed that “Some variables are born discrete, some achieve discreteness, and some have discreteness thrust upon ‘em” (cf. Staunton, 1860/1983, p. 1003; *Twelfth Night*, Act II, Scene 5). A variable might be considered “born discrete” if its conception, measurement, and essential nature were discrete or categorical in form. One instance of such a variable is treatment assignment in an experiment. In a simple experiment, participants are assigned randomly to a treatment or a control condition, and assignment to condition is recorded as a discrete score (e.g., 0 = control, 1 = treatment), with numeric assignment indicating group membership, not “more or less” of anything. Gene SNPs, mentioned earlier, are also candidate variables that can be considered to be “born discrete.” For example, the 5HTTLPR SNP has alleles characterized as either short (*s*) or long (*l*). Because a person inherits one of these alleles from the mother and one from the father, an individual can be characterized as *ss*, *sl*, or *ll*. Because the *s* allele has been found to confer more environmental susceptibility, the 5HTTLPR SNP is often scored discretely as 0, 1, or 2, indicating the number of *s* alleles at that particular location on the genome.

Ethnic group status, a component of many CR indices, is another example of a variable that might be considered “born discrete” (or naturally discrete), at least at first glance. Families in the Sameroff et al. (1987) study were identified as being of White, Black, or Puerto Rican ethnicity. Participants from the latter two ethnic groups were identified as at relatively higher risk relative to White participants, given discrimination and segregation that often accompany being a member of a disadvantaged or underrepresented group. Hence, a dichotomous score of 0 = White, 1 = non-White, was used as one component of the CR index. Whether a discrete classification into mutually exclusive ethnic groups is currently optimal or will be optimal in the future, given the mixed ethnicity of many individuals, is beyond the scope of the present paper to consider in detail, but will gain in importance in the future.

As for variables that “achieve discreteness,” father absence from the home may be one example. Suppose a researcher desired an index of father involvement in a child’s life, with lower levels of paternal involvement typically associated with increased risk for negative child outcomes. If father involvement were measured as, for example, hours per day the father interacts with the child, the researcher could easily leave the variable scored in quantitative form. However, presence (= 0) or absence (= 1) of the father or father figure in the home may be simpler to measure and is an obvious, if imprecise proxy for low levels of father involvement with the child and, thus, greater risk of negative developmental outcomes.

Turning to variables that have discreteness imposed on them, several variables used by Sameroff et al. (1987) are of this form. For example, mothers in the Sameroff et al. study completed three anxiety scales. Scores on these scales were standardized and summed, and the high-risk group was identified as the 25% of mothers with the highest anxiety scores. Clearly, a mother with a high level of anxiety almost certainly provides a less optimal caregiving environment for her child than a mother with a low level of anxiety. Scored as 0 (= low risk) vs. 1 (= high risk) does result in a variable for which a higher score conveys higher risk, although whether dichotomizing the quantitative form of the variable deserves critical appraisal.

### Continuous or quantitative indicators

Continuous indicators are ones that can take on a relatively large number of values with no “holes” in the number line. Height is one example. Height is typically measured discretely in a practical sense (e.g., to the nearest quarter inch), even though – in theory – all possible values between any two heights are admissible. Quantitative indicators are variables for which a higher score indicates more (or less) of a characteristic. If a scale has 10 items each answered on a 1-to-5 scale, the resulting sum score is, strictly, not continuous, as only integer values are allowed. Still, such a variable is a quantitative variable, with a higher score indicating more of what is assessed than a lower score.

Psychometric experts, including MacCallum, Zhang, Preacher, and Rucker (2002, Maxwell and Delaney (1993), and Preacher, Rucker, MacCallum and Nicewander (2005), have long decried dichotomizing continuous or quantitative scores. In general, it is unwise to dichotomize a quantitative indicator of risk, rather than leaving it in its quantitative form. Given limited space and the fact that risk indices analyzed below are discrete indicators, discussion of how to deal with quantitative indicators is beyond the scope of this paper.

### Problems of sample specificity

Currently, replicability of results across studies – more directly, lack of replicability of results across studies – is an issue of immense importance (Ioannidis, 2005; Simmons, Nelson, & Simonsohn, 2011). Efforts therefore should be made to ensure that comparable measurement operations and decisions are made across studies so that researchers can determine clearly whether research findings have been replicated. Evans et al. (2013) noted that standardizing scores to *M* = 0, *SD* = 1 is sample-specific and poses challenges when making comparisons across studies. This approach does place multiple variables in a given sample on the same scale, so they can be more reasonably composited into a risk index. But, if a particular sample were recruited from a high-risk subpopulation, a standardized mean of zero on *X* for that sample may have little comparability to a standardized mean of zero on *X* for a more representative sample from the population. Thus, converting to standardized scores within samples may destroy the ability to make informed comparisons across samples or studies.

The same criticism applies to the dichotomizing of scores, particularly given concerns about whether dichotomizing can be recommended. Sameroff et al. (1987) dichotomized several risk dimensions so the 25% of the sample with the most “risky” scores were given a score of 1, and the remainder of the sample was given a score of 0. But, the top 25% of a sample from a very high-risk subpopulation may represent a very different level of risk than the top 25% of a representative sample from the population. If researchers want to replicate results across studies, comparable measurement operations must be implemented so that risk in one sample can be compared informatively to risk in other samples.

## Interrogating or testing severely a risk index

### Testing hypotheses versus interrogating a model

The standard application of hypothesis testing in psychology, using a null hypothesis statistical test (NHST) approach, is to state a null hypothesis, H_0_, and a mutually exclusive and exhaustive alternative hypothesis, H_A_. For example, the null hypothesis might propose that all population regression coefficients in a regression equation are simultaneously zero, and the alternative hypothesis would be that at least one population regression coefficient is not zero. Data are collected, and a regression model is estimated, allowing a test of the null hypothesis. If the null hypothesis is rejected, the alternative hypothesis can be accepted, resulting in a confirmation of the hypothesis motivating the investigation. Hence, this approach has a bias in favor of confirmation, and only the theoretically uninteresting null hypothesis is tested.

A contrasting approach has been called *severe testing* by Mayo (2018), an approach positing that disconfirmation is the path forward in science. This approach is consistent with the ideas of Popper (1935/1959), Meehl (1990), and others that we should test our predictions, not null hypotheses, and test them as strongly or severely as possible. Under this approach, an investigator should develop a theoretical model for phenomena in a domain and then, after collecting data, test whether that model fits the data. When testing model predictions, this testing should involve severe testing of predictions or interrogating the fit of the model. If the model fits the data adequately, the model has survived a severe test and gained inductive support. Or, if the model is rejected as having poor fit to the data (i.e., model predictions are disconfirmed), something valuable has been learned – the theory was unable to account for the data, so requires modification to be in better accord with observations.

As applied to the construction of a CR index, the standard NHST approach might test whether a CR index formed as the sum of several risk indicators was a better predictor of a negative developmental outcome than was the single best indicator of risk. The contrasting, severe testing approach would test as directly and severely as possible whether equal weighting of risk indicators was acceptable or should be rejected in favor of a more complex and adequate form of weighting. If equal weighting cannot be rejected, the “equal weights” hypothesis has been interrogated and passed a severe test, supporting this simple weighting scheme.

### Summing indicators into a CR: The importance of metric

Once indicators of risk are identified, a researcher is confronted with the question of how to sum or combine multiple indicators into an index of CR. Evans et al. (2013) summarized typical approaches in the form of two options: First, if continuous risk indices are available and are in different metrics, one can standardize and sum the variables, although this makes sense only if risk indicators are correlated. Second, if risk indicators are not highly interrelated, researchers could dichotomize each indicator and then sum these into a cumulative index of risk.

Differing with Evans et al. (2013), I contend that the degree of correlation among risk indicators should play no role in deciding how to transform and sum variables. The sum of a set of indicators – quantitative or discrete, correlated or uncorrelated – can have substantial reliability in terms of stability over time and validity even if the sum has poor internal consistency (or homogeneity) reliability at a given point in time (Revelle & Condon, 2019).

The more important concern is the comparability of the metric of indicators. If one intends to sum a set of indicators, indicators must be on the same metric or approximately the same metric so indicators will have comparable contribution to the sum. The core criterion to satisfy for two measures to be considered to be on the same metric is that a one-unit increase on one variable is comparable or essentially identical to a one-unit increase on the other variable. Standardizing each of a set of quantitative indicators to *M* = 0, *SD* = 1 achieves a common metric across indicators, as a one-unit increase on any variable standardized in this fashion is a 1.0 *SD* increase. But, dichotomously scored variables, if scored in “0 vs. 1” fashion, also fall on a comparable metric across indicators, so the sum of such variables has ready interpretation. Once indicators are on the same metric, summing is reasonable and is often strongly recommended.

Indeed, summing a set of uncorrelated indicators can have advantages, such as leading to greater simplicity in analyses when predicting an outcome variable. Then, only a single predictor – the summed index of CR – is used as the representation of risk, so only a single regression weight for risk is estimated, rather than one regression weight for each of the separate indicators. To reiterate, the sum of a set of uncorrelated indicators may have substantial reliability in an “over time,” stability sense, and considerable validity, even if little internal consistency. So, regardless of the degree of correlation among risk indicators, the sum of indicators may have important benefits for analysis and interpretation.

### To weight differentially or not: Interrogating a weighting sccheme

Equal versus differential weighting of risk indicators when forming a sum is a key issue. If multiple risk indicators are on the same metric, summing the indicators may serve a legitimate scientific purpose regarding equal versus differential weighting of indicators, regardless of their degree of intercorrelation. Consider an outcome variable *Y* and two risk indicators, *X*_*1*_ and *X*_*2*_, which are assumed to be on the same metric (e.g., standardized to have equal means and equal variances, or both 0 – 1 dichotomies). A regression equation could be written as:

(1) $$Y_{i}=B_{0}+B_{1}X_{i1}+B_{2}X_{i2}+E_{i}$$

where *Y*_*i*_ is the score of person *i* on the outcome variable, *X*_*i*1_ and *X*_*i*2_ are scores of person *i* on the two risk indicators, respectively, *B*_0_ is the intercept, *B*_1_ and *B*_2_ are the raw score regression coefficients for the two risk indicators, respectively, and *E*_*i*_ represents error in predicting *Y* for person *i*. This equation would have a squared multiple correlation, or *R*^2^, that indicates the proportion of variance in *Y* accounted for by the weighted predictors. [Note: to ease presentation, the subscript *i* for person will be deleted from subsequent equations, with no loss in generality.].

Regardless of the degree of correlation between the two risk indicators, summing the two indicators would lead to the following equation:

(2) $$Y=B_{0}+B_{C}\left(X_{1}+X_{2}\right)+E^{*}$$

where *B*_*C*_ represents the raw score regression weight constrained to equality across the two risk indicators, E* represents the prediction error in this equation, and other symbols were defined above. Based on parameter nesting, Equation 2 is nested within Equation 1, as Equation 2 places an equality constraint on the two regression coefficients in Equation 1. Given the nesting, one could test the difference in explained variance for the two equations with the typical *F*-ratio for nested regression models (cf. Cohen, Cohen, West, & Aiken, 2003, p. 89), as:

(3) $$F_{\left(p_{1}-p_{2},N-p_{1}\right)}=\frac{\left(R_{Eq1}^{2}-R_{Eq2}^{2}\right)/\left(p_{1}-p_{2}\right)}{\left(1-R_{Eq1}^{2}\right)/\left(N-p_{1}\right)}$$

where *R*^2^_*Eq*1_ and *R*^2^_*Eq*2_ are squared multiple correlations under Equation 1 and Equation 2, respectively, *p*_1_and *p*_2_ are the number of regression slopes estimated in the two equations, respectively, and *N* is sample size. The resulting *F* ratio has (*p*_1_ – *p*_2_) and (*N* – *p*_1_) degrees of freedom and tests the hypothesis that the two regression slopes in Equation 1 are equal. If the *F* ratio were larger than the critical value at a pre-specified level (e.g., α= .05), the hypothesis of equality of regression coefficients could be rejected, and differential weighting of risk indicators is justified. On the other hand, if the *F* ratio did not exceed the critical value, the hypothesis of equality of regression coefficients cannot be rejected, and equal weighting is appropriate.

Note that Equations 1 and 2 easily generalize to situations with more than two risk indicators, but only if all indicators are on the same metric. If one had 10 risk indicators, an initial equation would have 10 predictors *X*_1_through*X*_10_ each with its own regression weight, as:

(4) $$Y=B_{0}+B_{1}X_{1}+B_{2}X_{2}+\ldots +B_{10}X_{10}+E$$

where symbols were defined above, and Equation 2 would then become:

(5) $$Y=B_{0}+B_{C}\left(X_{1}+X_{2}+\ldots +X_{10}\right)+E^{*}$$

where *B*_*C*_ represents the raw score regression weight constrained to equality across all 10 risk indices, and other symbols were defined above. The proper adaptation of Equation 3 would then provide an omnibus test of equality of the regression weights across all 10 risk indicators, an *F*-ratio that, in this case, would have 9 and (*N* – 11) degrees of freedom. If the resulting *F* ratio were nonsignificant, the hypothesis that the regression weights were simultaneously equal could not be rejected, justifying equal weighting of all 10 indicators when forming a CR index. Of course, if the *F* ratio were significant, the hypothesis of equality of regression weights would be rejectable, and a more informed and complex form of weighting might be considered. If equality of all regression weights were rejected, theory or the pattern of weights when all were freely estimated might offer options for more complex, but still restricted weighting schemes.

In evaluating comparisons such as those outlined in Equations 1 through 5, sample size and power to reject the hypothesis of equality of regression weights must be considered. As sample size increases, power to reject the “equal weights” hypothesis will increase. With extremely large sample size, an “equal weights” hypothesis might be rejectable via statistical test, even if the differences in the regression weights across predictors are trivial in magnitude. Hence, some notion of practical significance of the difference must also be weighed, whether of the magnitude of the differences in the raw score regression weights or the difference in the *R*^2^ values for the equations. Conversely, small sample size and resulting low power may lead to difficulty in rejecting the hypothesis of equality of regression weights even if these weights, in truth, differ in the population, essentially committing a Type II error. But, with small sample size, it may be more appropriate to proceed in the presence of a possible Type II error rather than promote differential weighting that cannot be justified statistically.

Equality versus differential weighting of predictors in regression analysis has surfaced as an issue with regularity over time (e.g., Wilks, 1938; Wainer, 1976; Dawes, 1979). Many have argued that equal weights are expected to lead to little loss in predictive accuracy relative to differential weights in many situations. Regrettably, this literature is beset with conflicting claims, much too voluminous to review here. In a different, though related vein, experts recently have discussed the fungibility, or exchangeability, of coefficients in regression (e.g., Waller, 2008) and structural equation models (e.g., Lee, MacCallum, & Browne, 2018). These researchers have cautioned against having too much faith in precise, optimal regression weights. For example, in a regression equation with three or more predictors, if a small drop in explained variance is allowed, an infinite number of different sets of regression weights all produce the same *R*^2^, and many of these sets of regression weights may have little similarity to the optimal least squares estimates in the equation that maximizes *R*^2^. Thankfully, if equal weighting of predictors is justified, the problem of fungible regression weights largely vanishes, as the number of regression weight estimates is reduced and the flexibility of the equation is curtailed.

In prior work on CR indices, equal weighting of risk indicators has virtually always been utilized. Equal weights may not be optimal in a least squares sense, but may be preferred on several grounds, including parsimony, efficiency, and openness to replication in subsequent studies. However, the issue of equal versus differential weighting of risk indicators should be interrogated or subjected to severe test, as this may impact the relations between a CR index and outcomes it should predict, so has a bearing on the construct validity of the CR index.

### Three guiding principles

When formulating and then interrogating a cumulative index derived from a number of risk indicators, the weighting and summing of indicators should be guided by justifiable analytic principles. Three principles are here proposed.

### Principle one: Do no harm overall

This principle represents the admonition that a model employing a CR index formed with equal weighting of risk indicators should not lead to a substantial reduction in model fit relative to that explained by a model with CR indicators having differential weights. A CR index can function as direct (or main) effect or as a component of an interaction when predicting a negative developmental outcome. If a model with an equally weighted CR index has fit similar to a model in which risk indicators have differential weights, no harm overall has occurred. But, if a model with an equally weighted CR index has worse fit than a model with differential weights for risk indicators, overall harm has occurred, and the a priori equal weighting of indicators into the CR index should be reconsidered and rejected.

### Principle two: Do no harm in particular

Here, the concern is with each individual component of a CR index. If risk indicators are equally weighted when forming an index of CR, some method should be used to determine whether the equal weighting has distorted the predictive effect of each risk indicator. If one or more risk indicators are compromised by the equal weighting, the formulation of the CR index should be reconsidered.

### Principle three: Do some good

As Evans et al. (2013) noted, the use of a CR index has a number of beneficial effects, including simplicity and reduction of potential multicollinearity if the components of the CR index had been used as separate, correlated predictors. Certainly, the use of a CR index will reduce the number of estimated regression slopes – a notable simplification – and may lead to improvements in other aspects of the equation, such as smaller standard errors of parameter estimates. If all or most of these improvements should occur, considerable good would have been bought by the use of the CR index.

## Example 1: Interrogating an environmental risk index

### Background

The study by Sameroff et al. (1987) was one of the first, if not the first, to use a CR index of environment risk for low intelligence. The sample consisted of 215 mothers and children, and child WPPSI Verbal IQ at age 4 years was the outcome variable. The 10 dichotomously scored risk factors are shown in Table 1. Certain variables (e.g., ethnic status, family support) were scored in direct and unambiguous fashion, and others (e.g., occupation, education) were dichotomized at commonly used points on their respective continua. For the remaining six variables, Sameroff et al. identified cut-scores that would leave about 25% of the sample with the highest risk receiving a risk score of 1, with the remaining 75% being assigned a risk score of 0. Additional details about each of the predictors were provided by Sameroff et al.

In Table 2, correlations among the 10 risk factors and the WPPSI Verbal IQ outcome variable are shown, along with estimated means and SDs of the variables (see Supplementary Material for how information from Sameroff et al., 1987, was used to develop Table 2). Inspection of Table 2 reveals that all 10 risk factors have negative correlations with WPPSI Verbal IQ, consistent with mean differences between low-risk and high-risk groups reported by Sameroff et al. The correlations in Table 2 also exhibit a strong positive manifold of correlations, with 44 of the 45 correlations among risk factors of positive valence. The single exception was the small negative correlation, *r* = −.03, between maternal mental health and ethnic status.

The risk factors shown in Table 1 are a varied amalgam of risk factors. The first four risk factors in Table 1 will hereinafter be called the Classic 4,^1^ given substantial research published over the past half century or longer on relations of these variables to child intelligence. From early work by Terman (1916) and Brigham (1923), down through work by Broman (1987; Broman et al., 1975), Jensen (1998) and Herrnstein and Murray (1994), and recently Johnson, Brett, and Deary (2010), researchers have studied relations of SES (occupation), education, and ethnicity with intelligence. The fourth indicator in the Classic 4 set – maternal interaction – is also based on substantial work. Yarrow and associates (e.g., Messer, Rachford, McCarthy, & Yarrow, 1987; Yarrow, MacTurk, Vietze, McCarthy, Klein, & McQuiston, 1984; Yarrow, Rubenstein, & Pedersen, 1975) reported strong relations between parental stimulation and interaction during a child’s infancy and young child problem-solving. Other work by Bradley and Caldwell and colleagues (Bradley & Caldwell, 1980; Bradley, Caldwell, & Elardo, 1977; Elardo, Bradley, & Caldwell, 1975) found strong relations (e.g., correlations ranging from .36 to .66) between observed mother-child interaction during the child’s infancy and child IQ at 3 years.

In contrast to the Classic 4, the remaining six risk variables, here referred to as the Modern 6, have received much less research attention with regard to predicting children’s intelligence. Certainly, father absence, high maternal anxiety, high numbers of difficult life events, and the remaining indicators likely confer risk for poorer development in general. But, these six indicators have received far less consistent attention as predictors of low child IQ as have the first four risk indicators.

### Regression analyses

Because the 215 observations had complete data on all variables, the summary data in Table 2 can be used to perform regression analyses. For all multiple regression analyses reported here, I used ordinary least squares (OLS) estimation in the PROC REG program in SAS (all analysis scripts are contained in Supplementary Material). To evaluate model fit, I used the *R* for a model and differences in *R*^2^ for competing models, the *F*-test for differences in fit of nested models, the adjusted *R*^2^ (*adj-R*^2^, which has a penalty for model complexity), and the Schwarz Bayesian Information Criterion (BIC).^2^ BIC values are not on a standardized metric, but lower values indicate better model fit.

### Replicating results reported by Sameroff et al.

Sameroff et al. (1987) reported that the single best predictor of Child Verbal IQ was occupation, *R*^2^ = .35. They noted that including all 10 risk factors led to a much higher level of explained variance, *R*^2^ = .51. Unfortunately, Sameroff et al. did not present any additional details, such as parameter estimates, and their *SE*s, for this 10-predictor model, so it is not possible to evaluate their reported results further.

I first wanted to replicate results reported by Sameroff et al. (1987) to verify that data in Table 2 were sufficient to reproduce their results. Model 1, shown in Table 3, used occupation as the sole predictor of child Verbal IQ and led to an *R*^2^ = .348. Adding the nine remaining risk factors led to Model 2 in Table 3, which had *R*^2^ = .519. Both of these models replicated closely *R*^2^ values reported by Sameroff et al. Note that, in Model 2, only the Classic 4 predictors had regression weights significant at *p* < .05, and none of the Modern 6 met this criterion.

### Interrogating weightings of all 10 indicators

Two a priori restricted models for the 10 risk indicators were candidates for interrogation or severe testing. The first model was one in which the regression weights for all 10 risk factors are constrained to equality. If one intended to employ an equally weighted sum of the 10 risk factors, as Sameroff et al. (1987) did later in their article, the resulting composite used as predictor in a regression model would lead to results that would be identical to a model with 10 predictors, but with regression weights for all 10 predictors constrained to equality. Given multicollinearity among risk indicators, constraining all regression weights to equality could have little effect on model fit, but deserves testing. The PROC REG program has an option for constraining regression weights; if constraints are imposed, tests of constraints or restrictions are also supplied^3^. Constraining all 10 regression weights to equality led to Model 3. Because 10 regression weights were constrained to equality, only a single regression weight was estimated, so 9 constraints or restrictions on weights were imposed. Results are shown in Table 3, which shows that Model 3 had an *R*^2^ = .442, a noticeable drop in explained variance relative to Model 2, Δ*R*^2^ = − .077, that was significant, *F*(9, 204) = 3.66, *p* = .0003. Some good was done, as the constrained estimate of the raw score regression coefficient, *B* = − 4.65, *SE* = 0.36, was accompanied by a much smaller standard error than was obtained by predictors in Model 2, but the overall drop in fit was troubling. Moreover, 8 of the 9 tests of constraints (see Restrictions 1 through 9 in Table 3) led to significant *t*-ratios (*p* < .05), suggesting that an equality constraint on regression coefficients across all 10 predictors led to too great a restriction on many of the regression coefficients. The upshot was that a model with all 10 regression weights constrained to equality, when tested severely, was rejected.

Given rejection of the first a priori model, the second a priori model to be interrogated was one in which the Classic 4 risk factors had regression weights constrained to equality, the Modern 6 risk factors had weights constrained to equality, but the weights for the Classic 4 and Modern 6 risk factors could differ. This model is termed Model 4, which had an *R*^2^ = .505. Model 4 is nested within Model 2 because it makes 8 fewer estimates, and the comparison with Model 2 represents a severe test of the highly restricted Model 4. For Model 4, the drop in explained variance relative to Model 2, Δ*R*^2^ = − .014, was very small in magnitude and not statistically significant, *F*(8, 204) = 0.74, *p* = .66. Moreover, as shown in Table 3, not one of the 8 constraint tests in Model 4 was statistically significant, supporting the contention that this pattern of constraints did not compromise any single regression weight estimate. The adjusted *R*^2^, *adj-R*^2^ = .500, for Model 4 was the highest adjusted *R*^2^ for any of the four models, attesting to the efficiency of Model 4 and its estimates. In addition, the BIC for Model 4 was lower than comparable BIC values for the first three models, implying Model 4 was the optimal model for the data. The superior fit of Model 4 relative to the more highly parameterized Model 2 represents superior return on investment in the efficient estimation of parameters, in the terms outlined by Rodgers (2019).

The regression coefficients and their SEs for Model 4 are shown in Table 3. The first four risk indicators had rather large coefficients, *B* = − 8.60, *SE* = 0.83, *p* < .0001, and the remaining six risk indicators had coefficients about one-sixth as large, *B* = − 1.43, *SE* = 0.71, *p* = .044, that just met the α= .05 criterion. So, all regression weights in Model 4 were statistically significant. Consider next the comparison of Models 3 and 4. In Model 4, the Classic 4 and Modern 6 risk indicators had different constrained estimates, whereas in Model 3 these estimates were constrained equal. Because the latter equality constraint led to a rather large and significant drop in fit, *F*(1, 212) = 27.21, *p* < .0001, this comparison supports the conclusion that the coefficients for Classic 4 and Modern 6 risk indicators in Model 4 differed significantly at *p* < .0001.

Comparisons among the four regression models satisfy the three principles set forth earlier. First, Model 4 did no harm overall, as it led to a small and nonsignificant drop in *R*^2^ relative to the most highly parameterized model, Model 2. Second, Model 4 did no harm in particular, because not one of the tests of parameter restrictions in Model 4 was significant. Thus, the constraints did not affect the ability of any one of the predictors to contribute to prediction of the outcome variable. Third, Model 4 did some good, as the *SE*s of parameter estimates were much reduced from the values estimated under Model 2, and all regression coefficients were statistically significant.

### Structural equation modeling

I conducted comparable analyses to those reported in Table 3 using structural modeling software (SEM) programs Mplus (Muthén & Muthén, 1998–2019) and lavaan (Rosseel, 2012) in R. Essentially identical results were obtained; given limitations of space, the full set of analysis scripts and descriptions of SEM results were placed in Supplementary Material.

### Discussion

The results of re-analyses of the Sameroff et al. (1987) data have two major implications, one more methodological and the other more substantive. The first, more methodological implication is that a single risk index created as the equally weighted sum of all risk factors may not be optimal for all domains of negative developmental outcome. That is, more differentiated summative indices of CR may have substantial analytic benefits. If researchers were to form the two separate unit-weighted CR indices suggested by the re-analysis, they could evaluate whether the same differential pattern of relations with child intelligence held in other samples. Many additional analytic options readily come to mind. For example, one could form a product of the two indices, representing an interaction of the Classic 4 and the Modern 6, to see if the effect of one of the CR indices varied as a function of the other. Thus, the effect of the CR index with larger predictive influence might be moderated by the number of risks to which the child was exposed comprised by the other CR index (e.g., father absence, high maternal anxiety, etc.).

The second, more substantive implication is that division into more than a single CR index might allow a clearer interpretation of results in the context of prior research. The re-analyses of Sameroff et al. (1987) data suggested the presence of two sets of risk factors, which I called the Classic 4 and Modern 6, for predicting child IQ. Importantly, the “Classic 4 vs. Modern 6” contrast of the 10 risk indicators may hold only for predicting child IQ or other forms of ability, such as achievement test scores or school grade point average. Developmentalists study a very broad array of consequential outcomes, including child mental health, conduct disorder problems, peer relations, attachment, and so forth. When researching these other domains of behavior, the Modern 6 might have predictive power that is equal to or even substantially stronger than the predictive power of the Classic 4. The relative predictive power of the two separate indices of CR across different domains of child and adolescent behavior might lead to more productive insights into relations between risk factors and child development than forcing all indicators into a single index that offers less flexibility in modeling.

## Example 2: Interrogating a genetic risk index

### Background

The second example is from Masarik et al. (2014), which used a CR index of genetic risk across five SNPs. Based on prior research investigating SNPs for environmental susceptibility, Masarik et al. designated the alleles as conferring susceptibility: (a) the short (*s*) allele of the 5-HTTLPR SNP in *5HTT*; (b) the A1 allele of the Taq1A polymorphism in *ANKK1* (often called DRD2 in prior work); (c) the 7R allele of exon-3 VNTR in *DRD4*; (d) the 10R allele of the 5’ VNTR in *DAT*; and (e) the Met allele of the Val158Met polymorphism in *COMT*. [For ease of presentation, the italicized acronyms for each allele are used in the remainder of this paper.].

Each of the five SNPs was originally assigned scores of 0, 1, or 2 based on how many of the plasticity alleles were observed. To enable more precise tests of possible forms of gene action, I created two redefined scores for each SNP, shown in Table 4. The *dominant* code reflects the assumption that only a single plasticity allele is needed to drive a genetic effect, and the presence of a second plasticity allele provides no additional risk or plasticity. The *recessive* code represents the hypothesis that no effect of the SNP would be seen unless two plasticity alleles are present. If the dominant and recessive codes are entered as separate predictors in an equation, several informative patterns might arise: (a) significant effect of the dominant code and near zero effect of the recessive code would support a conclusion that the SNP effect was dominant; (b) significant effect of the recessive code and near zero effect of the dominant code would support recessive gene action; and (c) equal regression weights for the dominant and recessive codes would support a conclusion of linear gene action.

The Masarik et al. (2014) investigation was of G×E interaction, and the environmental variable analyzed here was parental hostility toward a target adolescent when the adolescent was approximately 15 years of age. Parents and the adolescent engaged in videotaped interactions as they discussed several issues, including recent disagreements. Observers coded the videotaped interactions on 1-to-9 rating scales for three items – hostility, angry coercion, and antisocial behavior – of each parent toward the adolescent. Ratings were made for each parent and during each of two interaction tasks. The ratings were averaged across parents and across tasks to form a scale score for parental hostility, which could range from 1 to 9.

The outcome variable was obtained 16 years later when the target adolescents were now young adults averaging about 31 years of age. At this measurement point, each target individual was videotaped interacting with a romantic partner using similar methods as used for the prior parent-adolescent interactions. Observers coded these interactions using the same rating scales, which were again averaged into a scale for target hostility toward romantic partner.

Target participant sex (coded 0 = male, 1 = female) was used as a covariate, following Masarik et al. (2014). To allow comparisons of results across OLS regression and ML analyses using SEM software, analyses are restricted to the 281 participants who had complete data on the variables to be analyzed. For details on measures and procedures, see Masarik et al. (2014).

### Preliminary considerations

At least four concerns arise with analyzing genetic SNP effects in this data set. The first issue involves potential correlations of SNPs with other variables in the data set and among SNPs. Significant correlations of gene SNPs with variables such as parental hostility lead to difficulties in interpretation, such as passive gene-environment correlation. In Table 5, the correlations among five variables are shown: target (aged 31 years) hostility toward romantic partner, parental hostility toward target (aged 15 years), a gene SNP total score (sum across the 5 SNPs), a gene Dominant score (sum of the 5 dominant SNP scores), and a gene Recessive score (sum of the 5 recessive SNP scores). The *r* = .280 correlation of the first two variables is a strong inter-generational (i.e., parent behavior to offspring behavior) relation, particularly given the 16 years elapsed between the two measures. The correlations of target hostility with the three SNP scores were very small and nonsignificant, as were correlations of parental hostility with the SNP scores. The lack of any substantial correlation between gene SNP scores and environment scores simplifies theoretical and empirical considerations.

A second issue involves correlations among SNP scores, provided in Table 6. The first five variables in the table are the dominant scores for the five SNPs, and the remaining five variables are corresponding recessive scores. The five underscored coefficients in Table 6 are correlations between the dominant and recessive scores for each SNP. These correlations are *structurally positive* (i.e., necessarily positive) because one cannot have a score of 1 on the recessive score unless one has a 1 on the dominant score. These scores must correlate positively, and all were statistically significant (*ps* < .01), although not large. The other 40 correlations shown in Table 6 averaged almost precisely zero (−.003). Only one of these 40 correlations met the α = .05 criterion for significance, so the SNP scores were essentially independent.

The third issue is the form analyses might take. With a large number of SNP scores available, one could analyze (a) only a gene total score; (b) 5 linear (0,1,2) SNP scores; (c) only the 5 dominant SNP scores and/or their sum; (d) only the 5 recessive SNP scores and/or their sum; (e) the dominant and recessive total scores, or (f) 10 separate dominant and recessive SNP scores. For simplicity, only analyses using the gene total score and the 10 separate dominant and recessive SNP scores are reported here, emphasizing strengths and weaknesses of each approach. Parent hostility, the key environmental predictor, was mean-centered, to simplify interpretation.

A fourth issue is whether it is reasonable to form an equally weighted sum of a set of scores, here SNP scores, that are essentially uncorrelated. Based on homogeneity reliability theory, this makes no sense at all, as no increase in reliability would accompany the summing of uncorrelated scores. But, the equally weighted sum of a set of uncorrelated indicators might possess considerable reliability in terms of stability over time. Moreover, the sum can be used to test whether the risk indicators have significantly different weights in predicting an outcome. What is needed is a test of whether this equality constraint is reasonable or whether it costs too much in predictive power, and such tests are demonstrated in the following section.

### Regression analyses

Four regression models were fit using the genetic total score as the Gene variable, with parent hostility as Environment variable, to predict target hostility with romantic partner. The first model, Model 1 in Table 7, employed Female as control variable and used the E and G main effects as predictors. In Model 1, only Female and the Environment main effect were significant, and the overall model had a fairly large *R*^2^ = .1589. Adding the G×E interaction resulted in Model 2, which exhibited a moderately higher *R*^2^ = .1734. The increase in explained variance, Δ*R*^2^ = .0145, was significant, *F*(1, 276) = 4.87, *p* = .03, and the regression coefficient for the G×E interaction, *B* = 0.10 (*SE* = 0.05), *p* = .03, was significant and in the predicted direction.

Belsky and colleagues (Belsky, Pluess, & Widaman, 2013; Belsky & Widaman, 2018; Widaman, Helm, Castro-Schilo, Pluess, Stallings, & Belsky, 2012) contrasted strong and weak versions of differential susceptibility theory predictions and advocated comparative testing of these versions. Under the strong version, the Environment main effect should be zero in persons with no plasticity alleles; under the weak version, the Environment main effect might be non-zero even in persons with no plasticity alleles. To test whether the strong version was supported in this data set, I restricted the Environment main effect to zero in Model 3. As seen in Table 7, the drop in *R*^2^ was miniscule, Δ*R*^2^ = −.001, and not significant, *F*(1, 276) = 0.33, *p* = .56, and the regression coefficient for the G×E interaction, *B* = 0.07 (*SE* = 0.01), was significant, *t*(276) = 5.45, *p* < .0001. The improved level of statistical significance for the G×E interaction was the result of the much smaller *SE* for the interaction coefficient in Model 3 (*SE* = 0.01) relative to the comparable value in Model 2 (*SE* = 0.05). As seen in Table 7, the test of the restriction of the E main effect to zero did not approach significance, *t*(276) = − 0.58.

I fit one final model, Model 4, which added a restriction that the G main effect was zero. Because the E main effect was mean-centered, this represents a test that the G effect was zero at the mean of the E predictor. As seen in Table 7, the drop in explained variance moving from Model 3 to Model 4 was small, Δ*R*^2^ = −.0006, and not significant, *F*(1, 277) = 0.23, *p* = .63, and the test of the restriction was nonsignificant, *t*(277) = − 0.48. The regression coefficient for the G×E interaction remained unchanged in Model 4, a model in which the G×E interaction explained about half of the 17 percent of variance explained by the equation. The adjusted *R*^2^ value for Model 4 was higher than comparable values for other models, and the BIC value was lower than for other models, attesting to the efficiency and optimality of Model 4.

### Interrogating the weighting of SNPs

Models 1 through 4 utilized an equally weighted sum of the 5 SNP scores, so implicitly assumed that SNPs did not differ in their effects. This approach may have masked differential effects of the SNPs, so four additional regression models were tested, using the 5 dominant and 5 recessive SNP scores as separate predictors. In these analyses, the Gene main effect was represented by 10 separate dichotomous predictors, and the G×E interaction was represented by 10 product vectors, where each product vector was the product of a SNP score and parent hostility. With this approach, it becomes possible to interrogate or test more severely the equal weighting of SNPs.

The first of these models, identified as Model 5 in Table 8, used Female as control variable, parent hostility as Environment variable, and the 10 SNP scores to represent the main effect of Genes; this model explained over 17 percent of the variance in the outcome variable, *R*^2^ = .1755. As in the comparable Model 1 (Table 7), the E main effect was significant, *B* = 0.31 (*SE* = 0.07), *p* < .0001, but none of the 10 SNP scores met the .05 level of significance.

Model 6 added the 10 product vectors comprising the G×E interaction (see Table 8). Compared with Model 5, Model 6 explained more variance, *R*^2^ = .2128, but this increase in explained variance, Δ*R*^2^ = .0373, was nonsignificant, *F*(10,258) = 1.22, *p* = .28. Inspection of parameter estimates and their *SE*s in Table 8 reveals that not one of the G main effects had an effect significant at *p* < .05, and only one of the 10 G×E product terms (for the dominant effect of DAT) barely met the .05 level. In short, Model 6 looks like a veritable sea of nonsignificance.

However, restrictions on model parameter estimates can test whether coefficients differed from values that were predicted a priori. Model 7 invoked three restrictions: (a) due to presence of estimates for a G×E interaction, the main effect of the E variable was fixed at zero, (b) coefficients for the 10 gene SNP main effects were constrained to equality, and (c) coefficients for the 10 G×E interaction vectors were constrained to equality. In Table 8, Model 7, with *R*^2^ = .1724, explained less variance than Model 6, but the drop in fit, Δ*R*^2^ = −.0404, was not significant, *F*(19, 258) = 0.70, *p* = .82. This *F*-ratio is an overall or setwise test of the 19 restrictions in Model 7 and supports the contention that the 19 restrictions did no harm overall (or as a set). To ensure that no harm was done in particular, tests of parameter restrictions in Model 7 are shown in Table 9. There, the first restriction is the restriction of the E main effect to zero, Restrictions 2 – 10 reflect the equality constraint on the 10 SNP main effect coefficients, and Restrictions 11 – 19 reflect the equality constraint on the 10 G×E interaction coefficients. Not one of the tests of individual restrictions was significant at the .05 level

The final model, Model 8, added to Model 7 a constraint that the gene main effect coefficients be fixed at zero (see Table 8). Model 8 had a slightly lower level of explained variance, a drop in explained variance, Δ*R*^2^ = −.0006, that was neither large nor significant, *F*(1, 277) = 0.23, *p* = .63. To ensure that no harm was done to any single estimate, tests of restrictions in Model 8 are presented in Table 9. Not one of the 20 restrictions in Model 8 neared statistical significance, all *ps* > .22, so no single estimate was unduly harmed.

Note that Models 7 and 8 in Table 8 are identical in all ways – explained variance, parameter estimates and their SEs, adjusted *R*^2^ – to Models 3 and 4, respectively (cf. Table 7). Models 3 and 4 employed the Gene total score, so implicitly forced equality constraints on main and interactive effects of the gene SNP variables assuming that gene effects were (a) linear for each SNP and (b) equal in magnitude across SNPs. Models 7 and 8 enforced these constraints explicitly. The mathematical identity of all characteristics of Models 3 and 7 and of Models 4 and 8 is a brute force demonstration that the same end is achieved if one employs an a priori equally weighted sum of a set of indicators versus if one employs the indicators as separate predictors but forces equality constraints that embody those used in the a priori summation. Model 7, with its 19 restrictions, arrived at the same point as Model 3, but allowed the data to “complain” if any of the individual restrictions harmed the ability of any one of the genetic main or interaction components to contribute to the model. In effect, tests of restrictions in Model 7 allowed individual SNP components to “call foul” if a given constraint was overly restrictive. That not a single restriction was associated with statistical significance is a telling outcome that justifies the restrictions on a one-by-one basis. Similar comments apply to the identity between Models 4 and 8, as both of these models added the constraint that the gene main effect was zero.

In sum, Model 8 satisfies the three principles laid out early in the paper. First, Model 8 did no harm in general. It explained less variance than the most highly parameterized model, Model 6, but decreases in explained variance were nonsignificant. Second, Model 8 did no harm in particular, as not one of the 20 restrictions imposed on Model 8 led to any indication that a restriction harmed the contribution of any individual model component. Third, Model 8 did considerable good, with much smaller *SE*s of estimates relative to Model 6, a larger adjusted *R*^2^ than Models 5, 6, and 7, the lowest (i.e., best) BIC value of any of the models, and explicit justification of implicit assumptions made by Model 4. Thus, Model 8 reflects optimal return on investment when estimating regression parameters (cf. Rodgers, 2019).

### Structural equation modeling

I conducted comparable analyses to those reported in Table 8 using SEM programs Mplus (Muthén & Muthén, 1998–2019) and the lavaan package (Rosseel, 2012) in R. Essentially identical results were obtained; given limitations of space, the full set of analysis scripts and descriptions of SEM results were placed in Supplementary Material.

### Discussion

Results of the re-analyses of the Masarik et al. (2014) data have several implications for research using CR indices when investigating G×E interaction hypotheses. First, researchers can and should evaluate constraints implicitly imposed when a genetic risk index is used. If an equally weighted sum of gene SNPs is to be used, I showed how to test explicitly such equality constraints. Jolicoeur-Martineau, Belsky, Szekely, Widaman, Pluess, Greenwood, and Wazana (2020) proposed a way to differentially weight a small number of gene SNPs, and others have used GWAS results to differentially weight an extremely large number of gene SNPs (e.g., see Belsky & Harden, 2019). Regardless of whether equal or differential weighting of SNPs is used, researchers should adopt methods that test the optimality of the weights against models that relax the a priori constraints on weights. Successful demonstration that relaxing constraints does not improve results provides positive support for the more restricted sets of weights.

Second, analyses of individual genetic SNPs may be problematic, given the restricted range of scores for such variables. Individual SNPs have scores that fall on only a three-point scale (i.e., scored 0, 1, 2). Individual items on a personality scale or ability test scored on a three-point scale would never be expected to have strong or consistent relations with other criteria, so personality and ability items are typically summed into scale or test scores when predicting outcomes. Why research investigating gene main effects or G×E interactions has often been conducted with single SNP scores, given their restricted range and resulting very low statistical power, is an open question. Using some form of index of genetic CR is analogous to computing a personality scale score across several items. The items included can “borrow strength” from the other items comprising the sum, even if the individual indicators are uncorrelated, as was the case for the gene variables in this study. The methods proposed in this paper can be used to evaluate whether a priori weights used in computing the sum are justified.

A third implication is that one should use a confirmatory approach to model fitting and model comparison, rather than the exploratory approach that has been the standard for decades. If one used the typically taught approach to testing interactions, one would concentrate on the test of the G×E interaction when it is entered into the model. When analyzing the Masarik et al. (2014) data, this approach would have led to a decision that the interaction barely met the .05 level. But, the comparative approach of contrasting the fit of a priori models advocated by Belsky and colleagues challenges researchers to compare models that differ in their theoretical formulation. Under the strong form of differential susceptibility theory, the environment main effect should be nil for individuals with no plasticity alleles when a G×E interaction is included in the model. When such a constraint was invoked in the current analyses, the G×E interaction went from marginal significance to a very strong effect. Many research studies may have failed to “find” a significant G×E interaction due to the use of the low-powered, “recommended” way to test interactions. Failures to detect G×E interaction effects that are non-zero in the population amount to Type II errors, and such inadvertent failures may be legion.

A fourth and perhaps most important implication of results presented here is that prior behavior genetic (BG) and GWAS studies of behavioral outcomes may have followed research agendas that are ultimately of questionable intent or utility. Virtually all GWAS studies and most BG studies investigate only genetic main effects. The typical GWAS study uses an extremely large number of gene SNPs, perhaps a million or more, to determine whether each individual SNP is related to a given outcome, but main or interactive effects involving the environment are never included or tested. If the process generating the data included a G×E interaction with a strong differential susceptibility form, any genetic main effect would be nil or approximately nil at the mean of any key environmental variable, provided representative samples of persons and their typical environments are included. A nil genetic main effect in the presence of a G×E interaction does not mean that genes are unimportant in accounting for the outcome variable, but does imply that a robust effect of genes may be identified only if one simultaneously accounts for environmental effects in the form of a G×E interaction. *Failure to include environmental main and G×E interaction effects in GWAS studies may represent a search for the proverbial needle in a metaphorical haystack, when, in fact, no needle is present to be found.*

To circumvent this problem, a GWAS team might consider adding a measure of the environment into GWAS analyses. But, how one includes and models the E main effect and G×E interaction effect across a million or more SNPs become issues fraught with problems. Using the standard GWAS approach, the team might perform one million analyses – one for each SNP – each of which would include G and E main effects and a G×E interaction, with extremely careful control of Type I error rates. Or, they might decide to perform a million analyses with the E main effect fixed at zero, consistent with differential susceptibility model predictions. However, given the low power of such tests, this might be a dubious approach. The Masarik et al. (2014) data revealed G×E interaction components across just five SNPs that appeared to result in a veritable sea of nonsignificance when tested individually, but these became a robust G×E interaction once constraints across the G×E effects were imposed. The lack of significance of tests of those constraints lends substantial credibility to the equally weighted form of the constraints.

## General discussion

Developmental researchers have used risk indices in a number of different forms and done so quite profitably. Some investigators have used risk indices that were formed as the sum of multiple, dichotomously scored risk indicators, whereas others have summed standardized scores on more quantitative, continuous variables into indices of CR. Certain basic ways to evaluate validity of risk indices have been proposed. For example, if groups at different levels of a CR index have mean values on an outcome variable that are roughly linear, with higher risk associated with elevated problematic outcomes, this has very reasonably been touted as one form of evidence in support of the empirical and construct validity of the index of CR.

One goal of the current study was to propose ways to interrogate or test more severely (Mayo, 2018) the validity of an index of CR within an individual study, including analytic advice on how to perform more incisive analyses. If a researcher uses an equally weighted sum of risk indicators, it behooves that researcher to investigate whether the equality of the weights does any harm to analytic results. Methods proposed in this paper supplement prior work on equal weighting, providing model comparisons to test weighting schemes more informatively. Equal weighting may work well in most circumstances, but researchers should be made aware if equal weighting of all indicators does any harm to the modeling of data, and the methods proposed here support this goal of more informed and informative severe testing.

In many cases, equality of weights will be justified, as in analyses of the Masarik et al. (2014) data. In those analyses, the modeling provided no indication at all that forcing equal weights was problematic in any way and demonstrated the basis for the notable improvements associated with the highly constrained CR index. In contrast, analyses of the Sameroff et al. (1987) data provided evidence that a single unit-weighted sum of all 10 risk indices was not optimal. But, even here, the analysis did not imply that all 10 indicators should be used as separate, and separately weighted, indices of risk. Instead, the analysis suggested that two unit-weighted indices of CR would be optimal, slightly more complex than the use of a single unit-weighted composite, but far, far simpler than using 10 separate indicators.

Linear regression models with OLS estimation and SEM models with ML estimation led to very similar results. Because similar results were obtained using SAS PROC REG and SEM software, researchers should be encouraged initially to use analytic approaches with which they are most familiar and then to cross-validate results across multiple analytic approaches increase confidence in implications of their model comparisons.

Indices of environmental and genetic CR have been used profitably for many years, and the use of such indices has a large number of benefits and strengths, noted by Evans et al. (2013) and Belsky and Harden (2019), among others. Accompanying those strengths are a number of potential weaknesses that deserve investigation. The approaches advocated in the current paper are meant to supplement the evaluation of indices of CR, so that more informative use of such indices can occur. Failure to test the optimality of a priori compositing of risk indicators can mask important features of data, because non-optimal compositing of risk indices may lead to a failure to identify most accurately the underlying processes generating the data. Interrogating CR indices, as offered here, satisfies both the severe testing of models advocated by Mayo (2018) and the careful attention to return on investment when fitting models, with regard to both overall model fit and with regard to each parameter estimate, as promoted by Rodgers (2019). The future remains bright for research applications of indices of CR, supported by more self-critical, severely tested appraisal of their formulation and use.

## Supplementary Material

1

2

## Figures and Tables

**Table 1.** Ten Risk Factors Used by Sameroff et al. (1987)

	Risk Status	

Risk Variable	Low Risk (= 0)	High Risk (= 1)

Occupation	Skilled or higher	Semiskilled or lower

Education	High school graduate	High school non-graduate

Ethnic status	White	Non-White

Interaction	75% most spontaneous	25% least spontaneous

Mental health	0 – 1 contact	2 or more contacts

Family size	1 – 3 children	4 or more children

Life events	75% with fewest	25% with most

Perspectives	75% least controlling	25% most controlling

Family support	Father present	Father absent

Anxiety	75% least anxious	25% most anxious

*Note*: Tabled material is adapted from Table 2 of Sameroff et al. with minor modification.

**Table 2.** Correlations and Descriptive Statistics for Child Verbal IQ and 10 Risk Variables from Sameroff et al. (1987)

	Variable										

Variable	1. VIQ	2. Occ	3. Educ	4. Eth	5. Inter.	6. Ment	7. Fam	8. Life.	9. Pers	10. Sup	11. Anx

1. WPPSI VIQ	1.00										

2. Occupation	−.59	1.00									

3. Education	−.56	.62	1.00								

4. Ethnic	−.51	.53	.49	1.00							

5. Interaction	−.39	.22	.21	.15	1.00						

6. Mental health	−.16	.25	.28	−.03	.17	1.00					

7. Family size	−.36	.31	.39	.25	.34	.26	1.00				

8. Life events	−.26	.21	.22	.04	.17	.25	.25	1.00			

9. Perspectives	−.50	.53	.54	.57	.17	.09	.34	.22	1.00		

10. Fam. support	−.30	.33	.30	.38	.14	.19	.19	.08	.18	1.00	

11. Anxiety	−.24	.32	.24	.11	.14	.49	.15	.28	.13	.23	1.00

Mean	102.0	.20	.40	.39	.25	.40	.18	.25	.25	.25	.25

SD	18.0	.400	.490	.488	.433	.490	.384	.433	.433	.433	.433

*Note*: *N* = 215. The correlations in the table above have been re-arranged from those reported by Sameroff et al. (1987). As explained in the Appendix, three risk factor variables were reverse scored, and the correlation between WPPSI Verbal IQ and Occupation of *r* = −.59 as reported in text of the Sameroff et al. article was used, replacing the *r* = −.58 in their Table 3.

**Table 3.** Alternative Regression Models for the Sameroff et al. (1987) Data

	Model 1			Model 2		
	
Variable	*df*	*B* (*SE*)	*t* ^a^	*df*	*B* (*SE*)	*t* ^a^

Intercept	1	107.31 (1.11)	96.57	1	113.98 (1.44)	79.14

Occupation	1	− 26.55 (2.49)	−10.66	1	− 10.82 (3.12)	−3.46

Education				1	− 6.92 (2.52)	−2.74

Ethnic				1	− 6.17 (2.52)	−2.45

Interaction				1	− 9.38 (2.18)	−4.31

Mental health				1	2.05 (2.20)	0.93

Family size				1	− 1.82 (2.67)	−0.68

Life Events				1	− 3.78 (2.21)	−1.71

Perspectives				1	− 4.02 (2.77)	−1.45

Support				1	− 1.57 (2.28)	−0.69

Anxiety				1	− 1.77 (2.43)	−0.73

Restriction 1						

Restriction 2						

Restriction 3						

Restriction 4						

Restriction 5						

Restriction 6						

Restriction 7						

Restriction 8						

Restriction 9						

*R* ^2^		.348			.519	

Adjusted *R*^2^		.345			.496	

*F*(*ν*_1_,*ν*_2_) ^b^		113.74 (1, 213)			22.03 (10, 204)	

BIC ^c^		1160.61			1143.49	

	Model 3			Model 4		
		
Variable	*df*	*B* (*SE*)	*t* ^a^	*df*	*B* (*SE*)	*t* ^a^

Intercept	1	115.12 (1.37)	84.22	1	114.91 (1.29)	89.04

Occupation	1	− 4.65 (0.36)	− 12.98	1	− 8.60 (0.83)	− 10.38

Education	1	− 4.65 (0.36)	− 12.98	1	− 8.60 (0.83)	− 10.38

Ethnic	1	− 4.65 (0.36)	− 12.98	1	− 8.60 (0.83)	− 10.38

Interaction	1	− 4.65 (0.36)	− 12.98	1	− 8.60 (0.83)	− 10.38

Mental health	1	− 4.65 (0.36)	− 12.98	1	− 1.43 (0.71)	− 2.02

Family size	1	− 4.65 (0.36)	− 12.98	1	− 1.43 (0.71)	− 2.02

Life Events	1	− 4.65 (0.36)	− 12.98	1	− 1.43 (0.71)	− 2.02

Perspectives	1	− 4.65 (0.36)	− 12.98	1	− 1.43 (0.71)	− 2.02

Support	1	− 4.65 (0.36)	− 12.98	1	− 1.43 (0.71)	− 2.02

Anxiety	1	− 4.65 (0.36)	− 12.98	1	− 1.43 (0.71)	− 2.02

Restriction 1	− 1	− 151.9 (53.1)	− 2.86	− 1	− 27.68 (44.1)	− 0.63

Restriction 2	− 1	− 272.5 (89.4)	− 3.05	− 1	4.93 (65.4)	0.08

Restriction 3	− 1	− 472.6 (125.4)	− 3.77	− 1	31.50 (68.4)	0.46

Restriction 4	− 1	− 614.8 (124.8)	− 4.92	− 1	119.16 (64.9)	1.84

Restriction 5	− 1	− 268.7 (104.5)	− 2.57	− 1	106.50 (77.8)	1.37

Restriction 6	− 1	− 227.1 (106.8)	− 2.13	− 1	19.03 (81.8)	0.23

Restriction 7	− 1	− 149.9 (93.2)	− 1.60	− 1	− 44.51 (83.7)	− 0.53

Restriction 8	− 1	− 265.2 (99.4)	− 2.67	− 1	− 19.50 (60.0)	− 0.33

Restriction 9	− 1	− 189.5 (72.3)	− 2.62			

*R* ^2^		.442			.505	

Adjusted *R*^2^		.439			.500	

*F*(*ν*_1_,*ν*_2_) ^b^		168.36 (1, 213)			108.14 (2, 212)	

BIC ^c^		1127.37			1106.79	

*Note*: *N* = 215. Tabled values are raw score regression coefficients, their *SE*s in parentheses, and associated *t*-ratios. The *R*^2^ is the squared multiple correlation; the adjusted *R*^2^ is the shrunken estimate of squared multiple correlation that adjusts for model complexity.

a The *t* ratio has *df* equal to *ν*_2_ for the *F* ratio for the equation, shown below. With 204 or more *df*, the critical *t* value at the .05 level is 1.98.

b For the *F* ratio for each model, *ν*_1_ = numerator degrees of freedom, and *ν*_2_ = error (or denominator) degrees of freedom.

c BIC is the Schwarz Bayesian Information Criterion.

**Table 4.** Dominant and Recessive Codes for an Individual SNP

Number of relevant alleles	Dominant code	Recessive code

0	0	0

1	1	0

2	1	1

**Table 5.** Correlations of Target and Parent Hostility with Target Gene Indexes, based on Masarik et al. (2014) Data

	T Host	P Host	T Gene Total	T Gene Dom	T Gene Rec

Target Hostility	1.000				

Parent Hostility	.280	1.000			

T Gene Total	− .046	− .024	1.000		

T Gene Dom	− .060	− .111	.807	1.000	

T Gene Rec	− .015	.073	.802	.295	1.000

Mean	3.486	3.630	4.381	3.192	1.189

SD	1.652	1.426	1.392	0.869	0.869

*Note*: *N* = 281. Target Hostility and T Host = Target Hostility toward Romantic Partner at Age 31 years, Parent Hostility and P Host = Parent Hostility toward Target Aged 15 years, T Gene Total = Target Gene Total Index, T Gene Dom = Target Gene Dominant Index, T Gene Rec = Target Gene Recessive Index. Correlations greater than ~ |.12| are significant at *p* < .05, correlations greater than ~ |.154| are significant at *p* < .01.

**Table 6.** Correlations among Five Gene Dominant and Recessive Codes for Target Adolescent, using Masarik et al. (2014) Data

	ANKK1d^a^	DRD4d	DATh	5HTTh	COMTh	ANKK1r	DRD4r	DATr	5HTTr	COMTr

ANKK1d	1.000									

DRD4d	− .149	1.000								

DATd	.005	.015	1.000							

5HTTd	.045	− .090	.089	1.000						

COMTd	.002	− .075	.001	− .064	1.000					

ANKK1r	.280	− .036	− .012	− .013	.050	1.000				

DRD4r	− .097	.180	− .064	.014	− .004	− .027	1.000			

DATr	.036	− .036	.320	.092	− .007	− .005	.065	1.000		

5HTTr	.092	− .018	.090	.310	− .085	.065	− .073	.038	1.000	

COMTr	− .034	.033	− .050	.032	.350	.046	.016	.027	− .037	1.000

Mean	0.342	0.359	0.925	0.754	0.811	0.039	0.018	0.559	0.228	0.345

SD	0.475	0.481	0.263	0.431	0.392	0.194	0.132	0.497	0.420	0.476

*Note*: *N* 281. Underscored correlations must be non-zero, given the construction of the dominant and recessive codes for individual SNPs. Correlations greater than ~ |.12| are significant at *p* < .05, correlations greater than ~ |.154| are significant at *p* < .01.

a In SNP names, d = dominant code, r = recessive code.

**Table 7.** Alternative Regression Models for Target Hostility using Total Gene Index for Masarik et al. (2014) Data

	Model 1			Model 2		
	
Variable	*df*	*B* (*SE*)	*t* ^a^	*df*	*B* (*SE*)	*t* ^a^

Intercept	1	3.07 (0.32)	9.48	1	3.11 (0.32)	9.64

Female	1	0.94 (0.18)	5.10	1	0.95 (0.18)	5.22

E (Par Hostility)	1	0.32 (0.06)	4.97	1	− 0.12 (0.21)	− 0.57

G CR Index	1	− 0.03 (0.07)	− 0.38	1	− 0.03 (0.07)	− 0.52

GxE Interaction				1	0.10 (0.05)	2.21

*R*^2^		.1589			.1734	

Adjusted *R*^2^		.1498			.1615	

*F*(*ν*_1_,*ν*_2_) ^b^		17.44 (3, 277)			14.48 (4, 276)	

BIC		255.07			255.80	

	*df*	Model 3	*t*	*df*	Model 4	*t*

Intercept	1	3.10 (0.32)	9.63	1	2.96 (0.14)	21.83

Female	1	0.94 (0.18)	5.20	1	0.95 (0.18)	5.25

E (Par Hostility)	1	0.00 (———)		1	0.00 (——)	

G CR Index	1	− 0.03 (0.07)	− 0.48	1	0.00 (——)	

GxE Interaction	1	0.07 (0.01)	5.45	1	0.07 (0.01)	5.46

Restriction 1	− 1	− 6.32 (11.0)	− 0.58	− 1	− 5.98 (11.0)	− 0.54

Restriction 2				− 1	− 16.87 (35.1)	− 0.48

*R*^2^		.1724			.1718	

Adjusted *R*^2^		.1635			.1658	

*F*(*ν*_1_,*ν*_2_) ^b^		19.24 (3, 277)			28.83 (2, 278)	

BIC		250.50			245.09	

*Note*: *N* = 281. E (Par Hostility) stands for “Environment main effect, Parental Hostility”; G CR Index stands for “Gene main effect, a CR Index formed as the sum of scores on 5 target genes”; and GxE interaction is the product of the environmental and gene main effects. Tabled values are raw score regression coefficients, their *SE*s in parentheses, and associated *t*-ratios. The *R*^2^ is the squared multiple correlation; the adjusted *R*^2^ is the shrunken estimate of the squared multiple correlation.

a The *t* ratio has *df* equal to *ν*_2_ for the *F* ratio for the equation, shown below.

b For the *F* ratio for each model, *ν*_1_ = numerator degrees of freedom, and *ν*_2_ = error (or denominator) degrees of freedom.

**Table 8.** Alternative Regression Models for Hostility using Individual SNP Codes for Masarik et al. (2014) Data

	Model 5			Model 6		
	
Variable	*df*	*B* (*SE*)	*t* ^a^	*df*	*B* (*SE*)	*t* ^a^

Intercept	1	3.19 (0.45)	7.12	1	3.46 (0.46)	7.48

Female	1	0.92 (0.19)	4.91	1	0.89 (0.19)	4.70

Parent Hostility	1	0.31 (0.07)	4.75	1	− 0.58 (0.32)	− 1.79

ANKK1d	1	0.06 (0.20)	0.29	1	0.01 (0.21)	0.07

DRD4d	1	0.01 (0.20)	0.04	1	0.04 (0.20)	0.18

DATd	1	− 0.11 (0.37)	− 0.28	1	− 0.26 (0.38)	− 0.67

5HTTd	1	0.11 (0.23)	0.50	1	0.07 (0.23)	0.28

COMTd	1	− 0.24 (0.26)	− 0.93	1	− 0.26 (0.26)	− 1.00

ANKK1r	1	− 0.35 (0.49)	− 0.72	1	− 0.26 (0.50)	− 0.52

DRD4r	1	0.35 (0.72)	0.49	1	0.02 (0.92)	0.02

DATr	1	− 0.15 (0.20)	− 0.75	1	− 0.19 (0.20)	− 0.95

5HTTr	1	− 0.26 (0.23)	− 1.12	1	− 0.26 (0.23)	− 1.11

COMTr	1	0.31 (0.21)	1.48	1	0.29 (0.21)	1.39

PHost × ANKK1d				1	0.09 (0.15)	0.58

PHost × DRD4d				1	0.10 (0.16)	0.62

PHost × DATd				1	0.62 (0.31)	1.99

PHost × 5HTTd				1	0.14 (0.18)	0.78

PHost × COMTd				1	0.25 (0.19)	1.36

PHost × ANKK1r				1	0.36 (0.34)	1.05

PHost × DRD4r				1	0.56 (0.59)	0.95

PHost × DATr				1	− 0.11 (0.14)	− 0.75

PHost × 5HTTr				1	0.10 (0.16)	0.63

PHost × COMTr				1	− 0.13 (0.15)	− 0.86

*R*^2^		.1755			.2128	

Adjusted *R*^2^		.1386			.1457	

*F*(*ν*_1_,*ν*_2_) ^b^		4.76 (12, 268)			3.17 (22, 258)	

BIC		300.19			343.56	

	Model 7			Model 8		
	
Variable	*df*	*B* (*SE*)	*t* ^a^	*df*	*B* (*SE*)	*t* ^a^

Intercept	1	3.10 (0.32)	9.63	1	2.96 (0.14)	21.83

Female	1	0.95 (0.18)	5.20	1	0.95 (0.18)	5.25

Parent Pos Eng	1	0.00 (——)		1	0.00 (——)	

ANKK1d	1	− 0.03 (0.07)	− 0.48	1	0.00 (——)	

DRD4d	1	− 0.03 (0.07)	− 0. 48	1	0.00 (——)	

DATd	1	− 0.03 (0.07)	− 0. 48	1	0.00 (——)	

5HTTd	1	− 0.03 (0.07)	− 0. 48	1	0.00 (——)	

COMTd	1	− 0.03 (0.07)	− 0. 48	1	0.00 (——)	

ANKK1r	1	− 0.03 (0.07)	− 0. 48	1	0.00 (——)	

DRD4r	1	− 0.03 (0.07)	− 0. 48	1	0.00 (——)	

DATr	1	− 0.03 (0.07)	− 0. 48	1	0.00 (——)	

5HTTr	1	− 0.03 (0.07)	− 0. 48	1	0.00 (——)	

COMTr	1	− 0.03 (0.07)	− 0. 48	1	0.00 (——)	

PHost × ANKK1d	1	0.07 (0.01)	5. 45	1	0.07 (0.01)	5. 46

PHost × DRD4d	1	0.07 (0.01)	5. 45	1	0.07 (0.01)	5. 46

	Model 5			Model 6		
	
Variable	*df*	*B* (*SE*)	*t* ^a^	*df*	*B* (*SE*)	*t* ^a^

PHost × DATd	1	0.07 (0.01)	5. 45	1	0.07 (0.01)	5. 46

PHost × 5HTTd	1	0.07 (0.01)	5. 45	1	0.07 (0.01)	5. 46

PHost × COMTd	1	0.07 (0.01)	5. 45	1	0.07 (0.01)	5. 46

PHost × ANKK1r	1	0.07 (0.01)	5. 45	1	0.07 (0.01)	5. 46

PHost × DRD4r	1	0.07 (0.01)	5. 45	1	0.07 (0.01)	5. 46

PHost × DATr	1	0.07 (0.01)	5. 45	1	0.07 (0.01)	5. 46

PHost × 5HTTr	1	0.07 (0.01)	5. 45	1	0.07 (0.01)	5. 46

PHost × COMTr	1	0.07 (0.01)	5.45	1	0.07 (0.01)	5.46

*R*^2^		.1724			.1718	

Adjusted *R*^2^		.1635			.1658	

*F*(*ν*_1_,*ν*_2_) ^b^		19.24 (3, 277)			28.83 (2, 278)	

BIC		250.50			245.09	

*Note*: *N* = 281.

a The *t* ratio has *df* equal to *ν*_2_ for the *F* ratio for the equation, shown below.

b For the *F* ratio for each model, *ν*_1_= degrees of freedom numerator, and *ν*_2_= degrees of freedom error (or denominator).

**Table 9.** Tests Restrictions on Regression Parameters in Models 7 and 8 for Masarik et al. (2014) Data

	Model 7			Model 8		
	
Variable	*df*	*B* (*SE*)	*t* ^a^	*df*	*B* (*SE*)	*t* ^b^

Restriction 1	− 1	− 6.32 (11.0)	− 0.58	− 1	− 5.98 (11.0)	− 0.54

Restriction 2	− 1	− 3.22 (4.74)	− 0.68	− 1	− 3.84 (4.91)	− 0.78

Restriction 3	− 1	− 1.06 (5.61)	− 0.19	− 1	− 1.86 (5.85)	− 0.32

Restriction 4	− 1	− 9.49 (11.7)	− 0.81	− 1	− 13.16 (14.0)	− 0.94

Restriction 5	− 1	− 18.9 (13.3)	− 1.42	− 1	− 24.69 (17.9)	− 1.38

Restriction 6	− 1	− 0.86 (12.9)	− 0.07	− 1	− 9.23 (21.6)	− 0.43

Restriction 7	− 1	− 1.19 (13.7)	− 0.09	− 1	− 11.68 (25.8)	− 0.45

Restriction 8	− 1	4.02 (12.4)	0.32	− 1	− 7.95 (27.8)	− 0.29

Restriction 9	− 1	− 1.31 (11.7)	− 0.11	− 1	− 14.38 (29.6)	− 0.49

Restriction 10	− 1	3.28 (9.21)	0.36	− 1	− 12.04 (33.1)	− 0.36

Restriction 11	− 1	4.98 (7.29)	0.68	− 1	− 16.87 (35.1)	− 0.48

Restriction 12	− 1	9.40 (8.86)	1.06	− 1	4.89 (7.28)	0.67

Restriction 13	− 1	− 5.80 (17.1)	− 0.34	− 1	9.33 (8.85)	1.05

Restriction 14	− 1	− 2.35 (19.3)	− 0.12	− 1	− 6.04 (17.1)	− 0.35

Restriction 15	− 1	− 24.1 (19.8)	− 1.22	− 1	− 2.32 (19.2)	− 0.12

Restriction 16	− 1	− 17.8 (21.0)	− 0.84	− 1	− 23.86 (19.8)	− 1.21

Restriction 17	− 1	− 7.89 (19.8)	− 0.40	− 1	− 17.83 (21.0)	− 0.85

Restriction 18	− 1	− 2.35 (14.7)	− 0.16	− 1	− 7.70 (19.7)	− 0.39

Restriction 19	− 1	0.21 (13.2)	0.02	− 1	− 2.54 (14.7)	− 0.17

Restriction 20					0.25 (13.2)	0.02

*Note*: *N* 281. The beta distribution was used to compute probability levels for all *t* ratios (see SAS 9.4).

a The *t* ratios in this column have 277 *df*; all *ps* > .15.

b The *t* ratios in this column have 278 *df*; all *ps* > .17.

## Footnotes

1 I used the terminology of “classical versus modern” to provide shorthand labels for sets of predictors. “Classic 4” refers to the four risk factors featured in countless publications and theorizing over the past half century or more when investigating relations to child intelligence. “Modern 6” refers to the remaining six risk factors, which have received more recent attention as risk factors for low intelligence, but still decidedly less systematic attention than the Classic 4.

2 The PROC REG procedure in SAS yields two different Bayesian information criteria. One is labeled BIC and is the Sawa Bayesian information criterion; the other is labeled SBC and is the Schwarz Bayesian information criterion. To maintain consistency of reference with structural modeling results, I report the SBC values under the more common BIC label.

3 When restrictions on parameter estimates are invoked, the PROC REG procedure in SAS introduces a Lagrangian parameter for each restriction. The parameter estimate and standard error for each restriction are not on scales comparable to those for the intercept and constrained regression coefficients and their *SE*s. However, the point estimate for a restriction, when divided by its *SE*, is reported as a *t*-statistic, with probability value calculated using the beta distribution. See SAS 9.4 documentation for additional details.
