# Supplementary material for: Interrogating the validity of cumulative indices of environmental and genetic risk for negative developmental outcomes
Source: Dev Psychopathol. Author manuscript; Available in PMC 2024 Feb 1. (PMC9189257; doi:10.1017/S0954579421001097)
Supplement: 1 [file NIHMS1729548-supplement-1.pdf]

CAMBRIDGE UNIVERSITY PRESS

EXCLUSIVE LICENCE TO PUBLISH ("LTP")

This LTP records the terms under which the article specified below will be published in **Development and Psychopathology (DPP)** (the "**Journal**"). The Journal is owned and published by the Chancellor, Masters, and Scholars of the University of Cambridge acting through its department **Cambridge University Press** of University Printing House, Shaftesbury Road, Cambridge CB2 8BS, UK (the "**Publisher**").

THE ARTICLE	*Please insert the full title of the article below.*
**Article Title*:**	Experiences of Adversity in Childhood and Adolescence and Cortisol in Late Adolescence (the "**Contribution**")

This LTP can be used where a Contribution has one or more authors. The sole author (or the lead author, if applicable) must complete the box below and sign this LTP on behalf of themself (and all other authors, if any).

LEAD AUTHOR'S DETAILS AND SIGNATURE			
**Full Legal Name*:**	Courtenay Lorraine Kessler (the "**Lead Author**")		
**Authority to sign:**	By signing this LTP, I confirm and agree that:<br>i. All information that I have entered into this LTP is correct at the time of signature.<br>ii. **EITHER**, I am the sole author and owner of the copyright in the Contribution and I agree to the terms and conditions in this LTP.<br>iii. **OR**, the copyright in the Contribution is jointly owned by me and the Author(s) *listed below* and I agree to (and am authorized by each Author to agree to) the terms of this LTP on behalf of all Authors;<br>iv. **AND**, no other person nor entity has any copyright interest in the Contribution.		
**Signature*:**	[signature]	**Date*:**	8/3/21

OTHER AUTHORS' DETAILS

*If the Contribution is written by **two or more** authors and the copyright in the Contribution is **jointly** owned by them – please enter the details of all other individuals who contributed to the authoring of the Contribution in this box.*

If necessary, please add any more authors at the end of this LTP.

Full Legal Name*	Email address*	Affiliation*	Country of residence*
Suzanne Vrshek-Schallhorn	smschal2@uncg.edu	UNC-Greensboro	USA
Susan Mineka	suemineka@gmail.com	Northwestern U.	USA
Richard E Zinbarg	rzinbarg@northwestern.edu	Northwestern U	USA
Michelle Craske	craske@psych.ucla.edu	UCLA	USA
Emma K Adam	ek-adam@northwestern.edu	Northwestern U.	USA

(the Lead Author and each individual listed here and at the end of this LTP is, individually and collectively, the "**Author**")

CAMBRIDGE EMPLOYEE	
*You **must** check this box and enter details, if applicable.*	☐ One or more Authors are **employed by** Cambridge University Press or are **related to** a Cambridge University Press employee. Please provide names and describe the relationship(s):

SUPPLEMENTARY MATERIALS	*Identify any additional materials to be published in association with the Contribution*
If the Author intends to submit or upload any additional materials for online publication in association with the Contribution, please indicate by checking the applicable boxes in this section.	☒ **NO,** Supplementary Materials will **not** be submitted or uploaded by the Author for publication/uploading in connection with the Contribution.<br>☐ **YES,** Supplementary Materials which have been entirely created by the Author ("Original SM") will be submitted to the Publisher for publication/uploading in connection with the Contribution.<br>☐ **YES,** Supplementary Materials which contain third-party materials ("Third-party SM") will be submitted to the Publisher for publication/uploading in connection with the Contribution and the Author shall include a prominent notice stating the licence terms under which those additional materials can be made available.<br>(the "**Supplementary Material**")

PLEASE NOTE: Amended/alternative versions of this LTP will not be accepted and may delay or prevent publication
