# Supplementary material for: Interrogating the validity of cumulative indices of environmental and genetic risk for negative developmental outcomes
Source: Dev Psychopathol. Author manuscript; Available in PMC 2024 Feb 1. (PMC9189257; doi:10.1017/S0954579421001097)
Supplement: 2 [file NIHMS1729548-supplement-2.docx]

Supplementary Materials

For

**Interrogating the Validity of Cumulative Indices**

**of Environmental and Genetic Risk for**

**Negative Developmental Outcomes**

**Preparing Sameroff et al. (1987) Data for Analyses . . . . . . . . . . . . . . . . . . . . 2**

**Structural Equation Modeling Analyses . . . . . . . . . . . . . . . . . . . . . . . . . . 5**

**Analyses of Sameroff et al. (1987) data . . . . . . . . . . . . . . . . . . . . . . 5**

**Analyses of Masarik et al. (2014) data . . . . . . . . . . . . . . . . . . . . . . 8**

**Computer Scripts for Sameroff et al. (1987) Empirical Example . . . . . . . . . . . . 11**

**SAS script for regression models . . . . . . . . . . . . . . . . . . . . . . . . . . 11**

**Mplus scripts for Sameroff et al. (1987) data . . . . . . . . . . . . . . . . . . . . 14**

**lavaan script for Sameroff et al. (1987) data . . . . . . . . . . . . . . . . . . . . 17**

**Computer Scripts for Masarik et al. (2014) Empirical Example . . . . . . . . . . . . . 19**

**SAS script for regression models . . . . . . . . . . . . . . . . . . . . . . . . . . 19**

**Mplus scripts for Masarik et al. (2014) data . . . . . . . . . . . . . . . . . . . . 25**

**lavaan script for Masarik et al. (2014) data . . . . . . . . . . . . . . . . . . . . 35**

**Notification:**

All data and program analysis scripts in an Open Science Framework folder at <https://osf.oi/mynwh/> so that interested readers can download all material easily and reproduce results reported in the manuscript.

This document contains details about adapting data from Sameroff et al. (1987) for analyses. Next, structural equation modeling results for testing equality of regression weights are shown. Then computer codes for regression and SEM testing of models for both the Sameroff et al. and Masarik et al. (2014) data are provided..

**Preparing Sameroff et al. (1987) Data for Analyses**

I addressed requests to both Professor Arnold Sameroff and Professor Ronald Seifer for the raw data that served as the core of the cumulative risk analyses in their study (Sameroff et al., 1987). Specifically, I asked if I could receive a small data set with *N* = 215 containing scores on the 10 risk variables (just the dichotomized versions would be fine) and the WPPSI score used as the primary outcome measure. Not surprisingly, as the original study was published over 30 years ago, neither Dr. Sameroff nor Dr. Seifer was able to locate and supply the data.

Therefore, I used summary results presented in Sameroff et al. (1987) to develop a data set to be used for analyses. Table 2 of Sameroff et al. provided means and *t* test values comparing the low risk and high risk groups on each of the 10 risk variables. These results indicated that the low risk group on each risk variable had a higher mean Verbal IQ than did the high risk group, as expected. This means that, if a risk variable were scored as 0 = low risk, 1 = high risk, each of the risk variables should correlate negatively with Verbal IQ.

**Correlations**: Table 3 from Sameroff et al., labeled here as Supplemental Table 1::

**Supplemental Table 1**

Correlations among Study Variables: Table 3 from Sameroff et al. (1987)

|  | 1 | 2 | 3 | 4 | 5 | 6 | 7 | 8 | 9 | 10 |
| --- | --- | --- | --- | --- | --- | --- | --- | --- | --- | --- |
| 1. WPPSI VIQ | -- |  |  |  |  |  |  |  |  |  |
| 2. Mental health | ‒.16 | -- |  |  |  |  |  |  |  |  |
| 3. Family size | ‒.36 | .26 | -- |  |  |  |  |  |  |  |
| 4. Life events | ‒.26 | .25 | .25 | -- |  |  |  |  |  |  |
| 5. Education | .56 | ‒.28 | ‒.39 | ‒.22 | -- |  |  |  |  |  |
| 6. Perspectives | .50 | ‒.09 | ‒.34 | ‒.22 | .54 | -- |  |  |  |  |
| 7. Support | ‒.30 | .19 | .19 | .08 | ‒.30 | ‒.18 | -- |  |  |  |
| 8. Occupation | ‒.58 | .25 | .31 | .21 | ‒.62 | ‒.53 | .33 | -- |  |  |
| 9. Minority | ‒.51 | ‒.03 | .25 | .04 | ‒.49 | ‒.57 | .38 | .53 | -- |  |
| 10. Anxiety | ‒.24 | .49 | .15 | .28 | ‒.24 | ‒.13 | .23 | .32 | .11 | -- |
| 11. Interaction | .39 | ‒.17 | ‒.34 | ‒.17 | .21 | .17 | ‒.14 | ‒.22 | ‒.15 | ‒.14 |

One thing to note about Supplemental Table 1 above (Table 3 in Sameroff et al., 1987) is that variables 5, 6, and 11 (Education, Perspectives, and Interaction, respectively) were reported by Sameroff et al. as correlating positively with Verbal IQ. This suggests that, for correlations reported in this table, variables 5, 6, 11 had inadvertently been scored inversely, as 0 = high risk, 1 = low risk. Reverse scoring a variable reverses the sign (or valence) of the correlations of that variable with all other variables in the matrix. Reverse scoring variables 5, 6, and 11 led to a “Revised Table 3” from Sameroff et al., which is shown below:

**Supplemental Table 2**

Revised Table 3 from Sameroff et al. (1987), with Variables 5, 6, and 11 Reverse Scored

|  | 1 | 2 | 3 | 4 | 5 | 6 | 7 | 8 | 9 | 10 |
| --- | --- | --- | --- | --- | --- | --- | --- | --- | --- | --- |
| 1. WPPSI VIQ | -- |  |  |  |  |  |  |  |  |  |
| 2. Mental health | ‒.16 | -- |  |  |  |  |  |  |  |  |
| 3. Family size | ‒.36 | .26 | -- |  |  |  |  |  |  |  |
| 4. Life events | ‒.26 | .25 | .25 | -- |  |  |  |  |  |  |
| 5. Education | **‒.56** | **+.28** | **+.39** | **+.22** | -- |  |  |  |  |  |
| 6. Perspectives | **‒.50** | **+.09** | **+.34** | **+.22** | .54 | -- |  |  |  |  |
| 7. Support | ‒.30 | .19 | .19 | .08 | **+.30** | **+.18** | -- |  |  |  |
| 8. Occupation | ‒.58 | .25 | .31 | .21 | **+.62** | **+.53** | .33 | -- |  |  |
| 9. Minority | ‒.51 | ‒.03 | .25 | .04 | **+.49** | **+.57** | .38 | .53 | -- |  |
| 10. Anxiety | ‒.24 | .49 | .15 | .28 | **+.24** | **+.13** | .23 | .32 | .11 | -- |
| 11. Interaction | **‒.39** | **+.17** | **+.34** | **+.17** | .21 | .17 | **+.14** | **+.22** | **+.15** | **+.14** |

*Note*: Boldfaced correlations highlight the altered signs of correlations obtained after reverse scoring variables 5, 6, and 11.

In Supplemental Table 2 (immediately above), all 10 risk variables correlate negatively with WPPSI Verbal IQ scores, as they should, given the means reported in Table 2 of Sameroff et al. (1987). Also of note, after the reverse scoring, a virtual positive manifold of the 45 correlations among the 10 risk variables is evident, with only a single small negative correlation (*r* = ‒.03, between minority status and risk for mental health problems). The positive manifold of correlations implies that persons who have one risk factor tend to have other risk factors, although the strength of these correlations varies widely across risk factors.

The one final alteration I made to the correlation matrix shown as Table 2 in the current manuscript is the correlation between Occupation and the WPPSI Verbal IQ score. As shown in Table 3 of Sameroff et al. (1987), this correlation is listed as r = ‒.58. However, in text of the Sameroff et al. article, a value of r = ‒.59 was reported. When this latter value was used in analyses, I was able to replicate accurately analytic results reported in Sameroff et al.

**Means and standard deviations**: Next, means (*M*s) and standard deviations (*SD*s) for all variables had to be developed, because regression analyses using regression programs or structural modeling software require these statistics. Sameroff et al. (1987) did not list *M*s and *SD*s for the 11 variables, but did provide information in the text of the article that allowed reasonably close estimates of *M*s and *SD*s for variables. The 10 risk variables were dichotomous variables (scored 0 = low risk, 1 = high risk). It is well known that the *M* of a dichotomous variable is equal to the proportion, *p*, scoring 1, and the variance of such a variable is equal to *p*(1 – *p*), so the *SD* = *sqrt*[*p*(1 – *p*)], where *sqrt* is the “square root” operator.

The following provides information about how I estimated *M*s and *SD*s for the risk variables (with page numbers referring to pages in Sameroff et al., 1987):

**Mental health** (maternal): “… about 40% of the mothers in the sample was in the high-risk group” (p. 345). So, *M* = .40, *SD* = .490.

**Anxiety** (maternal): “The high-risk group contained the most anxious 25% of the sample” (p. 345). So, *M* = .25, *SD* = .433.

**Perspectives** (parental): “The high-risk group was the 25% who scored highest on the combined … dimensions” (p. 345). So, *M* = .25, *SD* = .433.

**Interaction** (mother interactive behaviors): “The high-risk group included the 25% least spontaneous mothers” (p. 345). So, *M* = .25, *SD* = .433.

**Education** (maternal): “Mothers with less than a high school education were considered high risk and comprised about 40% of the sample” (p. 345). So, *M* = .40, *SD* = .490.

**Occupation** (of head of household): “The high-risk group included families in which the head of household was unemployed, unskilled, or at best semiskilled and consisted of about 20% of the sample” (p. 345). However, earlier in the article (p. 344), 65 families were identified as being in the lowest socioeconomic groups, and 65 is 30% of 215. So, analyses were performed twice, once with *M* = .20, *SD* = .400; and once with *M* = .30, *SD* = .458. Because use of *M* = .20 and *M* = .30 had only minor effects on analyses, I reported results using *M* = .20, consistent with p. 345.

**Minority** (group status): “The high-risk group was formed from the minority status families (about 40%)” (p. 345). However, earlier in the manuscript (p. 344), Sameroff et al. noted that the sample included 79 Black families and 5 Puerto Rican families, and the 84 families in these two groups means that 39% of families were of minority status. I used this more precise value, so *M* = .39, *SD* = .488.

**Support** (family social support): “… family social support was defined simply as the presence or absence of a father in the home” (p. 345). Unfortunately, no percentage was given for the high-risk group. But, earlier in the article (p. 344), the authors had noted that “… 37 were single … and 17 were separated or divorced at some point during the course of the study. Summing these two groups gave 54 mothers, and 54 / 215 = .25. So, *M* = .25, *SD* = .433.

**Family size**: “Families with four or more children were placed in a high-risk group that consisted of about 20% of the sample” (p. 346). Earlier in the article (p. 344), the authors stated that 18 families had four children and 21 had 5 or more children. Thus, 39 families had four or more children, or 18% of the sample. I used this more precise value, so *M* = .18, *SD* = .384.

**Life events** (stressful): “The high-risk group consisted of the 25% of families who experienced the greatest number of stressful life events” (p. 346). So, *M* = .25, *SD* = .433.

For the outcome variable of WPPSI Verbal IQ, preliminary analyses of data from Table 2 of Sameroff et al. (1987) supports the suggestion that the mean IQ of the total sample was slightly above the population mean, and the nature of the sample, with inclusion of members from various risk groups implies the standard deviation was likely to be somewhat higher than in the population. As a result, I used M = 102, SD = 18.0. Little harm would arise if these values were not precisely accurate, as they serve as scaling constants on the intercept and raw score regression weights, respectively, so should not affect significance test values for the predictors in important ways.

**Structural Equation Modeling Analyses**

In developmental research, imposing and testing constraints on parameter estimates is uncommon using multiple regression modeling, but more common using structural equation modeling (SEM). Here, I report using SEM to demonstrate the essential identity of SEM results with the results using regression modeling. In this modeling, I used maximum likelihood (ML) estimation in Mplus (Muthén & Muthén, 1998-2017) and replicated results using the lavaan package in R (all program statements for these analyses are contained later in Supplementary Material). When evaluating model fit, I used: (a) the maximum likelihood *χ*^2^ test of model misfit, which is a test of perfect fit of model to data in the population, with a *p* < .05 providing a statistical basis for rejecting perfect fit of a model; (b) the root mean squared error of approximation (RMSEA) and its 90% CI, wherein a value less than .05 indicates close fit of model to data, values between .05 and .10 index increasingly poor fit, values over .10 imply unacceptable fit, and close fit cannot be rejected if the lower limit of the CI includes .05; (c) the comparative fit index (CFI) and Tucker-Lewis index (TLI), for both of which a value of .95 or higher indicates close fit of a model to data; and (d) the Schwarz Bayesian information criterion (BIC). To evaluate individual parameter estimates under constraint, I used modification indices (MIs). An MI is distributed approximately as a chi-squared variate with 1 degree of freedom (*df*), and estimates improvement in model fit that would accompany freely estimating the associated fixed or constrained parameter. Because the critical value of *χ*^2^(1) = 3.84 at α = .05, an MI larger than 3.84 implies model fit would improve if the parameter were freely estimated.

**Analyses of Sameroff et al. (1987) data.** I fit models identical to regression Model 1, with occupation as the only predictor, and Model 2, with all 10 risk indicators as separate predictors. These models are uninteresting from a model testing perspective using SEM because as many parameter estimates are made as sample statistics. As a result, both models had *χ*^2^ values of 0.0 with 0 degrees of freedom, so were not testable. Parameter estimates from these models are of interest, and they were virtually identical to the estimates for regression parameters in Models 1 and 2 shown in Table 3.

Next I fit a model identical to Model 3, constraining regression weights to invariance across all 10 risk indicators. Results for SEM Model 3 are shown in Supplemental Table 3. Because only a single regression coefficient estimate is made in Model 3, nine restrictions are invoked, leading to the 9 *df* for Model 3. As shown, Model 3 was rejectable statistically, *χ*^2^ (9) = 32.22, p < .001, the RMSEA was unacceptable (> .10), and both CFI and TLI were in the unacceptable range, < .85. Hence, Model 3 had very poor fit to the data. Interestingly, the estimates of regression weights and their *SE*s in Model 3 (in Supplemental Table 3) were virtually identical to the comparable estimates shown in Table 3 for regression analysis. Of equal interest is the estimates of MIs. Four of the 10 MIs were larger than 3.84, suggesting that a single equality constraint across all 10 regression parameter estimates was too stringent, harming the ability of some predictors to contribute accurately to prediction of the outcome variable.

I then fit an SEM model that was identical to regression Model 4, with one constraint on the parameter estimates across the first four predictors, and a second constraint on the parameter estimates for the last six predictors. Results for this Model 4 are shown in Supplemental Table 3. The overall fit of Model 4 was acceptable on all grounds, both statistically, with *χ*^2^ (8) = 6.27, *p* = .62, and practically, with RMSEA = 0.000, and both CFI and TLI = 1.00. In addition, Model 4 represented a substantial improvement in statistical fit over Model 3, Δ*χ*^2^ (1) = 25.95, p < .0001. Moreover, not one of the MIs approached significance, suggesting that the parameter constraints in Model 4 did not compromise the predictive power of any of the risk indicators.

Parameter estimates of regression coefficients and their *SE*s for SEM Model 4 are shown in Supplemental Table 3, and these are virtually indistinguishable from those reported in Manuscript Table 3 for regression Model 4. The only difference is the slightly smaller estimates of *SE*s under ML estimation in SEM programs, relative to the *SE*s under OLS estimation in regression analysis. [NOTE: The differences in *SE*s of parameter estimates occurs because ML estimation in SEM software uses a divisor of (*N* – 1) when estimating the variances of and covariances among regression estimates, whereas OLS estimation in regression software uses a divisor of (*N* – *p* – 1) , where *p* is the number of regression slopes estimated in a model. Use of a larger divisor under ML estimation leads to smaller estimated *SE*s of parameter estimates. Differences between *SE*s under ML and OLS estimation diminish as sample size increases and as the number of regression slopes estimated decreases.]

In sum, whether estimated using OLS estimation in regression packages or ML estimation in SEM programs, Model 4 satisfies the three principles set forth earlier in this paper: (1) Model 4, with its heavily restricted set of estimates, did no harm overall, as its overall fit to data was very good; (2) it did no harm in particular, as there was no indication that model constraints were too stringent; and (3) Model 4 did some good, leading to proper estimates of model parameters with substantially smaller *SE*s than for Model 2.

Supplemental Table 3

Alternative Structural Equation Models for the Sameroff et al. (1987) Data

|  |  | Model 3 | | | |  | Model 4 | | | | |
| --- | --- | --- | --- | --- | --- | --- | --- | --- | --- | --- | --- |
| Variable |  | *B (SE)* | *z* ^a^ | | MI ^b^ |  | *B* (*SE*) | | *z* ^a^ | | MI ^b^ |
| Intercept |  | 115.12 (1.36) | 84.62 | -- | |  | 114.91 (1.12) | 89.67 | | -- | |
| Occupation |  | ‒ 4.65 (0.36) | ‒ 13.04 | 8.25 | |  | ‒ 8.60 (0.82) | ‒ 10.45 | | 0.40 | |
| Education |  | ‒ 4.65 (0.36) | ‒ 13.04 | 3.58 | |  | ‒ 8.60 (0.82) | ‒ 10.45 | | 0.39 | |
| Ethnic |  | ‒ 4.65 (0.36) | ‒ 13.04 | 6.86 | |  | ‒ 8.60 (0.82) | ‒ 10.45 | | 0.22 | |
| Interaction |  | ‒ 4.65 (0.36) | ‒ 13.04 | 3.53 | |  | ‒ 8.60 (0.82) | ‒ 10.45 | | 0.22 | |
| Mental health |  | ‒ 4.65 (0.36) | ‒ 13.04 | 17.63 | |  | ‒ 1.43 (0.70) | ‒ 2.04 | | 3.42 | |
| Family size |  | ‒ 4.65 (0.36) | ‒ 13.04 | 0.44 | |  | ‒ 1.43 (0.70) | ‒ 2.04 | | 0.05 | |
| Life Events |  | ‒ 4.65 (0.36) | ‒ 13.04 | 1.08 | |  | ‒ 1.43 (0.70) | ‒ 2.04 | | 1.89 | |
| Perspectives |  | ‒ 4.65 (0.36) | ‒ 13.04 | 3.19 | |  | ‒ 1.43 (0.70) | ‒ 2.04 | | 1.17 | |
| Support |  | ‒ 4.65 (0.36) | ‒ 13.04 | 1.09 | |  | ‒ 1.43 (0.70) | ‒ 2.04 | | 0.14 | |
| Anxiety |  | ‒ 4.65 (0.36) | ‒ 13.04 | 6.93 | |  | ‒ 1.43 (0.70) | ‒ 2.04 | | 0.11 | |
|  |  |  |  | |  |  |  | |  | |  |
| *R*^2^ |  | .442 | | |  |  | .505 | | | |  |
| χ^2^ (**ν**) ^c^ |  | 32.22 (9) | | |  |  | 6.27 (8) | | | |  |
| prob |  | .0002 | | |  |  | .62 | | | |  |
| RMSEA (CI) |  | .110 (.070, .152) | | |  |  | .000 (.000, .068) | | | |  |
| CFI |  | .843 | | |  |  | 1.000 | | | |  |
| TLI |  | .825 | | |  |  | 1.000 | | | |  |
| BIC |  | 1742.89 | | |  |  | 1722.30 | | | |  |

*Note*: *N* = 215. ^a^ The *z* ratio is the ratio of the parameter estimate to its *SE*. ^b^ MI = modification index, in *χ*^2^ metric with 1 *df* ; critical value for 1 *df* at *p* = .05 is 3.84. ^c^ For the χ^2^ test statistic, **ν** = its degrees of freedom. RMSEA (CI) is the root mean square error of approximation, with its 90% CI in parentheses. CFI = comparative fit index. TLI = Tucker-Lewis index. BIC = Schwarz Bayesian Information Criterion.

**Analyses of Masarik et al. (2014) data**. Eight SEM models were fit to the Masarik et al. (2014) data, models that parallel the eight regression models reported in the manuscript. Given similarities between regression and SEM models for the Sameroff et al. (1987) data reported earlier, results for these models will be discussed in less detail because their results were very similar to regression results presented in Tables 7 and 8 in the manuscript. SEM Models 1 and 2 were saturated models, so had chi-square values of 0 with 0 df, and parameter estimates and *SE*s very similar to those for Models 1 and 2 shown in Table 7. SEM Models 3 and 4, paralleling regression Models 3 and 4, imposed, respectively, a constraint of the E main effect coefficient to zero and then additionally that the G main effect coefficient was zero. These restricted models had positive degrees of freedom. SEM Model 3 had a *χ*^2^(1) = 0.33, *p* = .56, and Model 4 had *χ*^2^(2) = 0.57, *p* = .75, indicating that the restrictions had no discernable negative effect on model fit to the data. Moreover, model estimates were essentially the same as for the corresponding regression models shown in Table 7.

SEM Models 5 and 6 were also saturated models, so had *χ*^2^ = 0 with 0 *df* and had parameter estimates and *SE*s similar to those for regression Models 5 and 6 shown in manuscript Table 8. SEM Model 7 imposed the same restrictions as those for regression Model 7, so the E main effect coefficient was fixed at zero, an equality constraint was placed across the 10 coefficients for the SNP main effects, and an equality constraint was placed across the 10 GxE coefficients. As shown in Supplemental Table 4, Model 7 had a non-significant level of statistical fit, *χ*^2^(19) =14.06, *p* = .14, so was not rejectable, and a very acceptable RMSEA of .000 [.000, .036], and very good CFI and TLI values of 1.000. Notably, MIs for constrained parameters did not come close to the critical value of 3.84, so there was no indication that restrictions were too strict.

The final model, Model 8, added the constraint that the gene main effect was zero. Fit of Model 8 was extremely good in all respects, with a non-significant statistical index of fit, *χ*^2^(20) =14.30, *p* = .78, perfect values of RMSEA of .000 [.000, .044], both CFI and TLI equal to 1.00, the best BIC value of all models, and non-significantly worse fit relative to Model 7, *χ*^2^(1) = 0.24, *p* = .83. Hence, the fit of Model 8 was extremely good. Notably, not one of the MIs for restricted estimates approached the critical value of 3.84, so there was no indication that any single restriction harmed the ability of any particular component to contribute to the model.

Because SEM Model 8 is essentially identical to regression Model 8 (cf. Table 8) and satisfies the modeling principles laid out earlier, it would be redundant here to belabor the superior nature of Model 8 relative to the less highly constrained models.

Supplemental Table 4

Alternative Structural Equation Models for the Masarik et al. (2014) Data

|  |  | Model 7 | | | |  | Model 8 | | |
| --- | --- | --- | --- | --- | --- | --- | --- | --- | --- |
| Variable |  | *B* (*SE*) | *z* ^a^ | MI ^b^ |  | | *B* (*SE*) | *z* ^a^ | MI ^b^ |
| Intercept |  | 3.10 (0.32) | 9.70 | -- |  | | 2.96 (0.14) | 21.95 | -- |
| Female |  | 0.95 (0.18) | 5.24 | -- |  | | 0.95 (0.18) | 5.28 | -- |
| Parent Hostility |  | 0.00 (----) | -- | 0.34 |  | | 0.00 (----) | -- | 0.30 |
| ANKK1d |  | ‒ 0.03 (0.06) | ‒ 0.48 | 0.00 |  | | 0.00 (----) | -- | 0.04 |
| DRD4d |  | ‒ 0.03 (0.06) | ‒ 0 .48 | 0.20 |  | | 0.00 (----) | -- | 0.10 |
| DATd |  | ‒ 0.03 (0.06) | ‒ 0.48 | 0.74 |  | | 0.00 (----) | -- | 0.95 |
| 5HTTd |  | ‒ 0.03 (0.06) | ‒ 0.48 | 0.22 |  | | 0.00 (----) | -- | 0.05 |
| COMTd |  | ‒ 0.03 (0.06) | ‒ 0.48 | 0.13 |  | | 0.00 (----) | -- | 0.25 |
| ANKK1r |  | ‒ 0.03 (0.06) | ‒ 0.48 | 0.47 |  | | 0.00 (----) | -- | 0.62 |
| DRD4r |  | ‒ 0.03 (0.06) | ‒ 0.48 | 0.43 |  | | 0.00 (----) | -- | 0.36 |
| DATr |  | ‒ 0.03 (0.06) | ‒ 0.48 | 0.59 |  | | 0.00 (----) | -- | 0.82 |
| 5HTTr |  | ‒ 0.03 (0.06) | ‒ 0.48 | 1.97 |  | | 0.00 (----) | -- | 1.20 |
| COMTr |  | ‒ 0.03 (0.06) | ‒ 0.48 | 2.86 |  | | 0.00 (----) | -- | 1.68 |
| PHost x ANKK1d |  | 0.07 (0.01) | 5.49 | 0.17 |  | | 0.07 (0.01) | 5.49 | 0.15 |
| PHost x DRD4d |  | 0.07 (0.01) | 5.49 | 0.43 |  | | 0.07 (0.01) | 5.49 | 0.45 |
| PHost x DATd |  | 0.07 (0.01) | 5.49 | 0.27 |  | | 0.07 (0.01) | 5.49 | 0.24 |
| PHost x 5HTTd |  | 0.07 (0.01) | 5.49 | 0.04 |  | | 0.07 (0.01) | 5.49 | 0.05 |
| PHost x COMTd |  | 0.07 (0.01) | 5.49 | 0.00 |  | | 0.07 (0.01) | 5.49 | 0.00 |
| PHost x ANKK1r |  | 0.07 (0.01) | 5.49 | 0.47 |  | | 0.07 (0.01) | 5.49 | 0.46 |
| PHost x DRD4r |  | 0.07 (0.01) | 5.49 | 0.68 |  | | 0.07 (0.01) | 5.49 | 0.69 |
| PHost x DATr |  | 0.07 (0.01) | 5.49 | 0.99 |  | | 0.07 (0.01) | 5.49 | 1.01 |
| PHost x 5HTTr |  | 0.07 (0.01) | 5.49 | 0.06 |  | | 0.07 (0.01) | 5.49 | 0.06 |
| PHost x COMTr |  | 0.07 (0.01) | 5.49 | 1.76 |  | | 0.07 (0.01) | 5.49 | 1.72 |
|  |  |  |  |  |  | |  |  |  |
| *R*^2^ |  | .172 | | |  | | .172 | | |
| χ**(ν**) ^c^ |  | 14.06 (19) | | |  | | 14.30 (20) | | |
| RMSEA [CI] |  | .000 [.000, .036] | | |  | | .000 [.000, .033] | | |
| CFI |  | 1.000 | | |  | | 1.000 | | |
| TLI |  | 1.000 | | |  | | 1.000 | | |
| BIC |  | 9338.38 | | |  | | 9332.98 | | |

*Note*: *N* = 281. ^a^ The *z* ratio is the ratio of the parameter estimate to its *SE*. ^b^ MI = modification index, in χ^2^ metric with 1 *df*. ^c^ For the χ^2^ test statistic, **ν** = its degrees of freedom.

**Computer Scripts for Sameroff et al. (1987) Empirical Example**

**SAS script for regression models**

options ls=**96** pageno=**1** pagesize=**100** decimal=**2**;

**data** sam87std (type=corr);

input _type_ $ **1**-**4** _name_ $ **7**-**14**

wppsi mental famsize lifevent educ perspect

support occupat minority anxiety interact ;

***********************************************************************************************;

* Data from Sameroff et al. (1987), published in Pediatrics ;

* ;

* Dependent variable ;

* wppsi Verbal IQ score on WPPSI, child age 4 years ;

* ;

* Risk factor definitions ;

* mental mother's poor mental health risk = more contacts with mental health system ;

* famsize family size risk = larger family size ;

* lifevent number of life events risk = more stressful life events (job loss,etc.);

* educ mother's education (reversed) risk = lower level of education ;

* perspect mother's rigidity, control risk = more rigid and controlling ;

* support social support (reversed) risk = father not in the home ;

* occupat occupation status (reversed) risk = unemployed, unskilled ;

* minority minority status risk = member of minority group ;

* anxiety anxiety risk = more anxious ;

* interact interaction style risk = low spontaneity, smiling, & vocalizing ;

* ;

* Categorical Predictors: risk factors coded 0 (low risk) vs. 1 (high risk) ;

* mental contacts with mental health 0 = 0 or 1 contact 1 = 2 or more ;

* famsize family size 0 = 1-3 children 1 = 4+ children ;

* lifevent number of life events 0 = 75% with fewest 1 = 25% with most ;

* educ mother's education 0 = high school grad 1 = not high school grad ;

* perspect mother's perspective 0 = 75% most flexible 1 = 25% most rigid ;

* support social support 0 = 0 or 1 contact 1 = 2 or more ;

* occupat occupation 0 = skilled 1 = semiskilled/unempl. ;

* minority minority status 0 = white 1 = non-white ;

* anxiety anxiety 0 = 75% least anxious 1 = 25% most anxious ;

* interact interaction style 0 = 75% most spontan. 1 = 25% least spontan. ;

***********************************************************************************************;

* occupat M = .20, SD = .40 because Method section said about 20 percent unemp or semiskl ;

***********************************************************************************************;

cards;

mean 102.00 0.40 0.18 0.25 0.40 0.25 0.25 0.20 0.39 0.25 0.25

std 18.00 .490 .384 .433 .490 .433 .433 .400 .488 .433 .433

N 215 215 215 215 215 215 215 215 215 215 215

corr wppsi 1.00 . . . . . . . . . .

corr mental -.16 1.00 . . . . . . . . .

corr famsize -.36 .26 1.00 . . . . . . . .

corr lifevent -.26 .25 .25 1.00 . . . . . . .

corr educ -.56 .28 .39 .22 1.00 . . . . . .

corr perspect -.50 .09 .34 .22 .54 1.00 . . . . .

corr support -.30 .19 .19 .08 .30 .18 1.00 . . . .

corr occupat -.59 .25 .31 .21 .62 .53 .33 1.00 . . .

corr minority -.51 -.03 .25 .04 .49 .57 .38 .53 1.00 . .

corr anxiety -.24 .49 .15 .28 .24 .13 .23 .32 .11 1.00 .

corr interact -.39 .17 .34 .17 .21 .17 .14 .22 .15 .14 1.00

;

**proc** **reg** data=sam87std(type=corr) ;

model wppsi = occupat / stb covb corrb ;

model wppsi = educ / stb covb corrb ;

model wppsi = minority / stb covb corrb ;

model wppsi = interact / stb covb corrb ;

model wppsi = mental / stb covb corrb ;

model wppsi = famsize / stb covb corrb ;

model wppsi = lifevent / stb covb corrb ;

model wppsi = perspect / stb covb corrb ;

model wppsi = support / stb covb corrb ;

model wppsi = anxiety / stb covb corrb ;

title 'Predicting WPPSI scores at 4 years from each separate risk index';

**run**;

**proc** **reg** data=sam87std(type=corr) outest=parms01 rsquare;

model wppsi = occupat / stb covb corrb aic sbc;

title 'Model 1: Predicting WPPSI scores at 4 years from Occupation';

**run**;

**data** parms01; set parms01; model = **01**;

**proc** **reg** data=sam87std(type=corr) outest=parms02 rsquare;

model wppsi = occupat educ minority interact

mental famsize lifevent perspect support anxiety / stb covb corrb aic sbc;

title 'Model 2: Predicting WPPSI scores at 4 years from 10 risk factors';

**run**;

**data** parms02; set parms02; model = **02**;

**proc** **reg** data=sam87std(type=corr) outest=parms03 rsquare;

model wppsi = occupat educ minority interact

mental famsize lifevent perspect support anxiety / stb covb corrb aic sbc;

restrict occupat = educ = minority = interact =

mental = famsize = lifevent = perspect = support = anxiety ;

title 'Model 3: Predicting WPPSI scores at 4 years from 10 risk factors -- ALL CONSTRAINED';

**run**;

**data** parms03; set parms03; model = **03**;

**proc** **reg** data=sam87std(type=corr) outest=parms04 rsquare;

model wppsi = occupat educ minority interact

mental famsize lifevent perspect support anxiety / stb covb corrb aic sbc;

restrict occupat = educ = minority = interact ,

mental = famsize = lifevent = perspect = support = anxiety ;

title 'Model 4: Predicting WPPSI scores at 4 years from 10 risk factors -- TWO CONSTRAINED SETS';

**run**;

**data** parms04; set parms04; model = **04**;

**data** fit;

set parms01 parms02 parms03 parms04;

deltaR2 = LAG(_rsq_) - _rsq_; if deltaR2 le **0** then deltaR2 = deltaR2 * -**1**;

numDF = LAG( _p_ ) - _p_ ; if numDF le **0** then numDF = numDF * -**1**;

denR2 = max(_rsq_ , lag(_rsq_));

denDF = min(_edf_ , lag(_edf_));

F = ((deltaR2 / numDF)) / ((**1** - denR2) / denDF);

prob = **1** - probF( F, numDF, denDF );

**proc** **print** data=fit;

var model _in_ _p_ _edf_ _rsq_ deltaR2 numDF denDF F prob _aic_ _sbc_;

title 'Sameroff: model comparisons for Models 00 - 04';

**run**;

**run**;

**quit**;

**Mplus scripts for Sameroff et al. (1987) data**

**Data set: file name is sameroff_summary.dat**

102.00 0.40 0.18 0.25 0.40 0.25 0.25 0.20 0.39 0.25 0.25

18.00 .490 .384 .433 .490 .433 .433 .400 .488 .433 .433

1.00

-.16 1.00

-.36 .26 1.00

-.26 .25 .25 1.00

-.56 .28 .39 .22 1.00

-.50 .09 .34 .22 .54 1.00

-.30 .19 .19 .08 .30 .18 1.00

-.59 .25 .31 .21 .62 .53 .33 1.00

-.51 -.03 .25 .04 .49 .57 .38 .53 1.00

-.24 .49 .15 .28 .24 .13 .23 .32 .11 1.00

-.39 .17 .34 .17 .21 .17 .14 .22 .15 .14 1.00

**Model 1 Script**

TITLE: Sameroff et al. (1987) data

run -- Model 1

Occupation only

DATA: FILE IS sameroff_summary.dat;

type is correlation means stdeviations;

nobservations = 215;

VARIABLE: NAMES ARE

wppsi

mental famsize lifevent educ control

support occupat minority anxiety interact ;

!MISSING = ALL . ;

USEVAR =

wppsi

occupat ;

ANALYSIS: ITERATIONS = 10000 ;

MODEL: wppsi on occupat ;

OUTPUT: SAMPSTAT STANDARDIZED TECH1 TECH4 ;

**Model 2 Script**

TITLE: Sameroff et al. (1987) data

run -- Model 2

All 10 predictors, separately weighted

DATA: FILE IS sameroff_summary.dat;

type is correlation means stdeviations;

nobservations = 215;

VARIABLE: NAMES ARE

wppsi

mental famsize lifevent educ control

support occupat minority anxiety interact ;

!MISSING = ALL . ;

USEVAR =

wppsi

occupat educ minority interact

mental famsize lifevent control support anxiety;

ANALYSIS: ITERATIONS = 10000 ;

MODEL: wppsi on occupat

educ

minority

interact

mental

famsize

lifevent

control

support

anxiety;

OUTPUT: SAMPSTAT STANDARDIZED TECH1 TECH4 ;

**Model 3 Script**

TITLE: Sameroff et al. (1987) data

run -- Model 3

10 predictors, all constrained equal

DATA: FILE IS sameroff_summary.dat;

type is correlation means stdeviations;

nobservations = 215;

VARIABLE: NAMES ARE

wppsi

mental famsize lifevent educ control

support occupat minority anxiety interact ;

USEVAR =

wppsi

occupat educ minority interact

mental famsize lifevent control support anxiety;

ANALYSIS: ITERATIONS = 10000 ;

MODEL: wppsi on occupat (B01)

educ (B01)

minority (B01)

interact (B01)

mental (B01)

famsize (B01)

lifevent (B01)

control (B01)

support (B01)

anxiety (B01);

OUTPUT: SAMPSTAT STANDARDIZED TECH1 TECH4 ;

**Model 4 Script**

TITLE: Sameroff et al. (1987) data

run -- Model 4

Classic 4 constrained equal

Modern 6 constrained equal

Classic 4 NOT equal to Modern 6

DATA: FILE IS sameroff_summary.dat;

type is correlation means stdeviations;

nobservations = 215;

VARIABLE: NAMES ARE

wppsi

mental famsize lifevent educ control

support occupat minority anxiety interact ;

USEVAR =

wppsi

occupat educ minority interact

mental famsize lifevent control support anxiety;

ANALYSIS: ITERATIONS = 10000 ;

MODEL: wppsi on occupat (B01)

educ (B01)

minority (B01)

interact (B01)

mental (B02)

famsize (B02)

lifevent (B02)

control (B02)

support (B02)

anxiety (B02) ;

OUTPUT: SAMPSTAT STANDARDIZED modindices(0) TECH1 TECH4 ;

**lavaan script for Sameroff et al. (1987) data**

#------------------------------------------------------------------------------------#

library(lavaan) # activate #

library(psych) # desired packages #

library(GPArotation) #

#------------------------------------------------------------------------------------#

# # set working directory, if needed #

# setwd('D:/Dropbox/.../sameroff/lavaan') # not needed for this script file #

#------------------------------------------------------------------------------------#

cor_mat = matrix(

c(

1.00, -.16, -.36, -.26, -.56, -.50, -.30, -.59, -.51, -.24, -.39,

-.16, 1.00, .26, .25, .28, .09, .19, .25, -.03, .49, .17,

-.36, .26, 1.00, .25, .39, .34, .19, .31, .25, .15, .34,

-.26, .25, .25, 1.00, .22, .22, .08, .21, .04, .28, .17,

-.56, .28, .39, .22, 1.00, .54, .30, .62, .49, .24, .21,

-.50, .09, .34, .22, .54, 1.00, .18, .53, .57, .13, .17,

-.30, .19, .19, .08, .30, .18, 1.00, .33, .38, .23, .14,

-.59, .25, .31, .21, .62, .53, .33, 1.00, .53, .32, .22,

-.51, -.03, .25, .04, .49, .57, .38, .53, 1.00, .11, .15,

-.24, .49, .15, .28, .24, .13, .23, .32, .11, 1.00, .14,

-.39, .17, .34, .17, .21, .17, .14, .22, .15, .14, 1.00 ),

nrow = 11, ncol = 11, byrow = TRUE )

cor_mat

sd_mat = diag(c( 18.00, .490, .384, .433, .490, .433, .433, .400, .488, .433, .433) )

sam.cov = sd_mat %*% cor_mat %*% sd_mat

sam.cov

rownames(sam.cov)= c('wppsi',

'mental' ,'famsize','lifevent','educ' ,'control',

'support','occupat','ethnic' ,'anxiety','interact')

colnames(sam.cov)= c('wppsi',

'mental' ,'famsize','lifevent','educ' ,'control',

'support','occupat','ethnic' ,'anxiety','interact')

sam.cov

sam.mn = matrix(c(102.00, 0.40, 0.18, 0.25, 0.40, 0.25, 0.25, 0.20, 0.39, 0.25, 0.25),

nrow = 1, ncol = 11, byrow = TRUE)

sam.mn

colnames(sam.mn)= c('wppsi',

'mental' ,'famsize','lifevent','educ' ,'control',

'support','occupat','ethnic' ,'anxiety','interact')

sam.mn

#------------------------------------------------------------------------------------#

# Sameroff et al. data using environmental CR index to predict child Verbal IQ #

#------------------------------------------------------------------------------------#

# IMPORTANT NOTE: USING sem FITTING FUNCTION IN lavaan #

#------------------------------------------------------------------------------------#

#------------------------------------------------------------------------------------#

# Model #1: Predict WPPSI Verbal IQ from Occupation alone #

#------------------------------------------------------------------------------------#

model01 = '

wppsi ~ B01*occupat

'

fit.mod01 = sem(model = model01, sample.cov = sam.cov, sample.mean = sam.mn,

sample.nobs = 215, fixed.x = FALSE)

summary(fit.mod01, fit.measures = TRUE, standardized = TRUE)

inspect(fit.mod01)

#------------------------------------------------------------------------------------#

# Model #2: Predict WPPSI Verbal IQ from all 10 separate risk factors #

#------------------------------------------------------------------------------------#

model02 = '

wppsi ~ B01*occupat + B02*educ + B03*ethnic + B04*interact +

B05*mental + B06*famsize + B07*lifevent + B08*control +

B09*support + B10*anxiety

'

fit.mod02 = sem(model = model02, sample.cov = sam.cov, sample.mean = sam.mn,

sample.nobs = 215, fixed.x = FALSE)

summary(fit.mod02, fit.measures = TRUE, standardized = TRUE)

inspect(fit.mod02)

#------------------------------------------------------------------------------------#

# Model #3: Predict WPPSI Verbal IQ from all 10 constrained risk factors #

#------------------------------------------------------------------------------------#

model03 = '

wppsi ~ B01*occupat + B01*educ + B01*ethnic + B01*interact +

B01*mental + B01*famsize + B01*lifevent + B01*control +

B01*support + B01*anxiety

'

fit.mod03 = sem(model = model03, sample.cov = sam.cov, sample.mean = sam.mn,

sample.nobs = 215, fixed.x = FALSE)

summary(fit.mod03, fit.measures = TRUE, standardized = TRUE, modindices = TRUE)

inspect(fit.mod03)

lavTestScore(fit.mod03, uni = TRUE)

#------------------------------------------------------------------------------------#

# Model #4: Predict WPPSI VIQ from Classic 4 & Modern 6, separately constrained #

#------------------------------------------------------------------------------------#

model04 = '

wppsi ~ B01*occupat + B01*educ + B01*ethnic + B01*interact +

B02*mental + B02*famsize + B02*lifevent + B02*control +

B02*support + B02*anxiety

'

fit.mod04 = sem(model = model04, sample.cov = sam.cov, sample.mean = sam.mn,

sample.nobs = 215, fixed.x = FALSE)

summary(fit.mod04, fit.measures = TRUE, standardized = TRUE, modindices = TRUE)

inspect(fit.mod04)

lavTestScore(fit.mod04, uni = TRUE)

**Computer Scripts for Masarik et al. (2014) Empirical Example**

**SAS Script for regression models**

options ls=**116** pageno=**1** pagesize=**100** nolabel;

libname in 'c:\users\kwidaman\dropbox\mystuff\kw\mss\mss_in_prep\widaman risk indices\data\april_data';

**data** april;

infile 'c:\users\kwidaman\dropbox\mystuff\kw\mss\mss_in_prep\widaman risk indices\data\april_data\april352.dat' missover;

input famid

female male

parwarm parhost tarwarm tarhost f14warm f14host

tankk tdrd4 tdat thtt tcomt

tankkd tdrd4d tdatd thttd tcomtd

tankkr tdrd4r tdatr thttr tcomtr

complet;

**proc** **means** data=april;

title 'descriptives on April''s N = 352 data set';

**run**;

**data** april; set april;

*------------------------------------------------------------------------------;

* variable description ;

* ;

* famid family id number ;

* female sex of child 0 = male, 1 = female ;

* male sex of child 1 = male, 0 = female ;

* ;

* parwarm parent positive engagement to target range 1.50 - 8.75 ;

* parhost parent hostility to target range 1.00 - 9.00 ;

* tarwarm target positive engagement to parents range 1.00 - 7.25 ;

* tarhost target hostility to parents range 1.00 - 9.00 ;

* f14warm target positive engagement to partner range 1.00 - 9.00 ;

* f14host target hostility to partner range 1.00 - 9.00 ;

* ;

* tankk target ANKK additive 0,1,2 mall. alleles ;

* tdrd4 target DRD4 additive 0,1,2 mall. alleles ;

* tdat target DAT additive 0,1,2 mall. alleles ;

* thtt target 5-HTTLPR additive 0,1,2 mall. alleles ;

* tcomt target COMT additive 0,1,2 mall. alleles ;

* ;

* tankkd target ANKK dominant, mall. alleles 0 = none, 1 = 1 or 2 ;

* tdrd4d target DRD4 dominant, mall. alleles 0 = none, 1 = 1 or 2 ;

* tdatd target DAT dominant, mall. alleles 0 = none, 1 = 1 or 2 ;

* thttd target 5-HTTLPR dominant, mall. alleles 0 = none, 1 = 1 or 2 ;

* tcomtd target COMT dominant, mall. alleles 0 = none, 1 = 1 or 2 ;

* ;

* tankkr target ANKK recessive, mall. alleles 0 = 0, 1, 1 = 2 ;

* tdrd4r target DRD4 recessive, mall. alleles 0 = 0, 1, 1 = 2 ;

* tdatr target DAT recessive, mall. alleles 0 = 0, 1, 1 = 2 ;

* thttr target 5-HTTLPR recessive, mall. alleles 0 = 0, 1, 1 = 2 ;

* tcomtr target COMT recessive, mall. alleles 0 = 0, 1, 1 = 2 ;

* ;

* complet complete data for analysis 0 = no, 1 = yes ;

*------------------------------------------------------------------------------;

*********************************************************************;

* create new predictors for centering ;

parwarmc = parwarm ;

parhostc = parhost ;

**proc** **standard** data=april mean=**0** out=april;

var parwarmc parhostc ;

title 'centering predictors on April''s N = 352 data set';

**run**;

**proc** **means** data=april;

**run**;

**quit**;

*********************************************************************;

* key variables for regression analyses ;

* female ;

* parwarm parhost f14warm f14host ;

* parwarmc parhostc ;

* tankk tdrd4 tdat thtt tcomt ;

* tankkr tdrd4r tdatr thttr tcomtr ;

* tankkd tdrd4d tdatd thttd tcomtd ;

*********************************************************************;

***************************************************************************************;

* HOSTILITY MODELS ;

***************************************************************************************;

**data** aprilw; set april;

if complet eq **1**;

*--------------------------------------------------------------------------------------;

* re-centering parwarm and parhost for N = 281 ;

*--------------------------------------------------------------------------------------;

**proc** **standard** data=aprilw mean=**0** out=aprilw;

var parwarmc parhostc;

title 'centering parental warmth and hostility for N = 281';

**run**;

**proc** **means** data=aprilw;

title 'descriptives on data for N = 281';

**run**;

**quit**;

**data** aprilw; set aprilw;

genetot = tankk + tdrd4 + tdat + thtt + tcomt ;

genedom = tankkd + tdrd4d + tdatd + thttd + tcomtd;

generec = tankkr + tdrd4r + tdatr + thttr + tcomtr;

*--------------------------------------------------------------------;

* product variables for CENTERED parental HOSTILITY by TARGET SNPs ;

*--------------------------------------------------------------------;

cphtankk = parhostc * tankk; cphtankkr = parhostc * tankkr; cphtankkd = parhostc*tankkd;

cphtdrd4 = parhostc * tdrd4; cphtdrd4r = parhostc * tdrd4r; cphtdrd4d = parhostc*tdrd4d;

cphtdat = parhostc * tdat ; cphtdatr = parhostc * tdatr ; cphtdatd = parhostc*tdatd ;

cphthtt = parhostc * thtt ; cphthttr = parhostc * thttr ; cphthttd = parhostc*thttd ;

cphtcomt = parhostc * tcomt; cphtcomtr = parhostc * tcomtr; cphtcomtd = parhostc*tcomtd;

phtankkd = parhostc * tankkd ;

phtdrd4d = parhostc * tdrd4d ;

phtdatd = parhostc * tdatd ;

phthttd = parhostc * thttd ;

phtcomtd = parhostc * tcomtd ;

phtankkr = parhostc * tankkr ;

phtdrd4r = parhostc * tdrd4r ;

phtdatr = parhostc * tdatr ;

phthttr = parhostc * thttr ;

phtcomtr = parhostc * tcomtr ;

phgentot = parhostc * genetot ;

**proc** **corr** data=aprilw ;

var f14host parhostc genetot genedom generec ;

title 'correlations among key variables';

**run**;

**proc** **corr** data=aprilw ;

var tankk tdrd4 tdat thtt tcomt ;

title 'correlations among genetic SNPs (0, 1, 2 scores)';

**run**;

**quit**;

**proc** **corr** data=aprilw alpha;

var tankk tdrd4 tdat thtt tcomt ;

title 'correlations among genetic SNPs (0, 1, 2 scores), plus coefficient alpha';

**run**;

**quit**;

**proc** **corr** data=aprilw ;

var tankkd tdrd4d tdatd thttd tcomtd

tankkr tdrd4r tdatr thttr tcomtr ;

title 'correlations among dominant and recessive SNP scores';

**run**;

**quit**;

**proc** **corr** data=aprilw alpha;

var tankkd tdrd4d tdatd thttd tcomtd

tankkr tdrd4r tdatr thttr tcomtr ;

title 'correlations among dominant and recessive SNP scores, plus coefficient alpha';

**run**;

**quit**;

**proc** **ttest** data=aprilw;

class female;

var f14host;

title 'sex difference on hostility at age 31 years';

**run**;

**quit**;

**proc** **corr** data=aprilw;

var f14host female parhostc genetot phgentot;

title 'correlations among key variables for regression analyses';

**run**;

**quit**;

************************************************************************************************;

* analyses using the genetot variable -- the sum of the five SNP values ;

************************************************************************************************;

**proc** **reg** data=aprilw outest=parms01 rsquare;

model f14host = female parhostc genetot

/ stb ss2 covb corrb aic bic sbc;

title1 'SUM 5 GENES: Model 01: three main effects only';

title2 ' ';

**run**;

**data** parms01; set parms01; model = **01**;

**proc** **reg** data=aprilw outest=parms02 rsquare;

model f14host = female parhostc genetot phgentot

/ stb ss2 covb corrb aic bic sbc;

title1 'SUM 5 GENES: Model 02: three main effects PLUS GXE INTERACTION';

**run**;

**data** parms02; set parms02; model = **02**;

**proc** **reg** data=aprilw outest=parms03 rsquare;

model f14host = female parhostc genetot phgentot

/ stb ss2 covb corrb aic bic sbc;

restrict parhostc = **0**;

title1 'SUM 5 GENES: Model 03: Strong Diff Susceptibility';

title2 'RESTRICT ENVIRONMENT MAIN EFFECT = 0';

**run**;

**data** parms03; set parms03; model = **03**;

**proc** **reg** data=aprilw outest=parms04 rsquare;

model f14host = female parhostc genetot phgentot

/ stb ss2 covb corrb aic bic sbc;

restrict parhostc = **0**,

genetot = **0**;

title1 'SUM 5 GENES (0-10): Model 04: Strong Diff Susceptibility';

title2 'RESTRICT ENV MAIN EFFECT & GENE MAIN EFFECT = 0';

**run**;

**data** parms04; set parms04; model = **04**;

**data** first;

set parms01 parms02 parms03 parms04;

deltaR2 = LAG(_rsq_) - _rsq_; if deltaR2 le **0** then deltaR2 = deltaR2 * -**1**;

numDF = LAG( _p_ ) - _p_ ; if numDF le **0** then numDF = numDF * -**1**;

denR2 = max(_rsq_ , lag(_rsq_));

denDF = min(_edf_ , lag(_edf_));

F = ((deltaR2 / numDF)) / ((**1** - denR2) / denDF);

prob = **1** - probF( F, numDF, denDF );

**proc** **print** data=first;

var model _in_ _p_ _edf_ _rsq_ deltaR2 numDF denDF F prob _aic_ _sbc_;

title1 'SUM 5 GENES (0-10): model comparisons for Models 01 - 04';

title2 ' ';

**run**;

***********************************************************************************************;

* analyses using 5 DOMINANT (gendom5) and 5 RECESSIVE (genrec5) separate indicators ;

***********************************************************************************************;

**proc** **reg** data=aprilw outest=parms5 rsquare;

model f14host = female parhostc

tankkd tdrd4d tdatd thttd tcomtd

tankkr tdrd4r tdatr thttr tcomtr

/ stb ss2 covb corrb aic bic sbc;

title1 '10 SNPs (@0-1): Model 5: three main effects only';

title2 ' ';

**data** parms5; set parms5; model = **5**;

**proc** **reg** data=aprilw outest=parms6 rsquare;

model f14host = female parhostc

tankkd tdrd4d tdatd thttd tcomtd

tankkr tdrd4r tdatr thttr tcomtr

cphtankkd cphtdrd4d cphtdatd cphthttd cphtcomtd

cphtankkr cphtdrd4r cphtdatr cphthttr cphtcomtr

/ stb ss2 covb corrb aic bic sbc;

title1 '10 SNPs (@0-1): Model 6: three main effects PLUS GXE INTERACTION';

title2 ' ';

**data** parms6; set parms6; model = **6**;

**proc** **reg** data=aprilw outest=parms7 rsquare;

model f14host = female parhostc

tankkd tdrd4d tdatd thttd tcomtd

tankkr tdrd4r tdatr thttr tcomtr

cphtankkd cphtdrd4d cphtdatd cphthttd cphtcomtd

cphtankkr cphtdrd4r cphtdatr cphthttr cphtcomtr

/ stb ss2 covb corrb aic bic sbc;

restrict parhostc = **0**;

restrict tankkr = tdrd4r = tdatr = thttr = tcomtr =

tankkd = tdrd4d = tdatd = thttd = tcomtd ;

restrict cphtankkr = cphtdrd4r = cphtdatr = cphthttr = cphtcomtr =

cphtankkd = cphtdrd4d = cphtdatd = cphthttd = cphtcomtd ;

title1 '10 SNPs (@0-1)s: Model 7 Strong Diff Sus -- ';

title2 'RESTRICT ENV = 0, RESTRICT G & GXE EFFECT TO EQUALITY';

**data** parms7; set parms7; model = **7**;

**proc** **reg** data=aprilw outest=parms8 rsquare;

model f14host = female parhostc

tankkd tdrd4d tdatd thttd tcomtd

tankkr tdrd4r tdatr thttr tcomtr

cphtankkd cphtdrd4d cphtdatd cphthttd cphtcomtd

cphtankkr cphtdrd4r cphtdatr cphthttr cphtcomtr

/ stb ss2 covb corrb aic bic sbc;

restrict parhostc = **0**;

restrict tankkr = tdrd4r = tdatr = thttr = tcomtr =

tankkd = tdrd4d = tdatd = thttd = tcomtd = **0** ;

restrict cphtankkr = cphtdrd4r = cphtdatr = cphthttr = cphtcomtr =

cphtankkd = cphtdrd4d = cphtdatd = cphthttd = cphtcomtd ;

title1 '10 SNPs (@0-1)s: Model 8 Strong Diff Sus --';

title2 'RESTRICT ENV & G = 0, & RESTRICT GXE EFFECT TO EQUALITY';

**data** parms8; set parms8; model = **8**;

**data** sixth;

set parms5 parms6 parms7 parms8;

deltaR2 = LAG(_rsq_) - _rsq_; if deltaR2 le **0** then deltaR2 = deltaR2 * -**1**;

numDF = LAG( _p_ ) - _p_ ; if numDF le **0** then numDF = numDF * -**1**;

denR2 = max(_rsq_ , lag(_rsq_));

denDF = min(_edf_ , lag(_edf_));

F = ((deltaR2 / numDF)) / ((**1** - denR2) / denDF);

prob = **1** - probF( F, numDF, denDF );

**proc** **print** data=sixth;

var model _in_ _p_ _edf_ _rsq_ deltaR2 numDF denDF F prob _aic_ _sbc_;

title1 '10 SNPs (@0-1): model comparisons for Models 5 - 8';

title2 ' ';

**run**;

**proc** **corr** data=aprilw outp=rmtx;

var tankkd tdrd4d tdatd thttd tcomtd

tankkr tdrd4r tdatr thttr tcomtr ;

title1 'correlations among dominant and recessive SNP scores';

**run**;

**quit**;

**data** rmtx; set rmtx;

if _type_ eq 'CORR';

keep tankkd tdrd4d tdatd thttd tcomtd

tankkr tdrd4r tdatr thttr tcomtr ;

**data** rmtx; set rmtx;

array cl[**10**] tankkd tdrd4d tdatd thttd tcomtd

tankkr tdrd4r tdatr thttr tcomtr ;

do i = **1** to **10**;

if cl[i] ge **0.17** then cl[i] = **.**;

end;

keep tankkd tdrd4d tdatd thttd tcomtd

tankkr tdrd4r tdatr thttr tcomtr ;

**proc** **print** data=rmtx;

title1 'correlations among dominant and recessive alleles';

**run**;

**proc** **iml**;

use rmtx;

read all var _num_ into a;

print a;

b = shape(a , **100** , **1**);

print b;

create corvec from b [colname={ 'correl' }];

append from b;

close corvec;

**quit**;

**proc** **means** data=corvec;

title1 'mean correlation among dominant and recessive alleles';

**run**;

**run**;

**quit**;

**Mplus Scripts for Masarik et al. (2014) Empirical Example**

**Model 1**

TITLE: SEM Model 1, using Total Gene index

sample size = 281

E main effect = parent HOSTILITY to target aged 15

G main effect = sum across 5 SNPs

GxE interact = not included

outcome = target HOSTILITY to partner aged 31

DATA: FILE IS april352.dat;

VARIABLE: NAMES ARE

famid

female male

parwarm parhost tarwarm tarhost f14warm f14host

tankk tdrd4 tdat thtt tcomt

tankkd tdrd4d tdatd thttd tcomtd

tankkr tdrd4r tdatr thttr tcomtr

complet;

USEVARIABLES are

f14host female parhostc genetot ;

MISSING are .;

USEOBSERVATIONS = complet eq 1;

DEFINE: parwarmc = parwarm - 4.4937722;

parhostc = parhost - 3.6304840;

genetot = tankk + tdrd4 + tdat + thtt + tcomt;

pwgentot = parhostc * genetot ;

phtankkd = parhostc * tankkd ;

phtdrd4d = parhostc * tdrd4d ;

phtdatd = parhostc * tdatd ;

phthttd = parhostc * thttd ;

phtcomtd = parhostc * tcomtd ;

phtankkr = parhostc * tankkr ;

phtdrd4r = parhostc * tdrd4r ;

phtdatr = parhostc * tdatr ;

phthttr = parhostc * thttr ;

phtcomtr = parhostc * tcomtr ;

ANALYSIS: iterations = 10000;

MODEL: f14host on female (B01)

parhostc (B02)

genetot (B03) ;

OUTPUT: SAMPSTAT MODINDICES(0) TECH1 TECH4 standardized residual;

**Model 2**

TITLE: SEM Model 2, using Total Gene index

sample size = 281

E main effect = parent HOSTILITY to target aged 15

G main effect = sum across 5 SNPs

GxE interact = estimated

outcome = target HOSTILITY to partner aged 31

DATA: FILE IS april352.dat;

VARIABLE: NAMES ARE

famid

female male

parwarm parhost tarwarm tarhost f14warm f14host

tankk tdrd4 tdat thtt tcomt

tankkd tdrd4d tdatd thttd tcomtd

tankkr tdrd4r tdatr thttr tcomtr

complet;

USEVARIABLES are

f14host female parhostc genetot phgentot;

MISSING are .;

USEOBSERVATIONS = complet eq 1;

DEFINE: parwarmc = parwarm - 4.4937722;

parhostc = parhost - 3.6304840;

genetot = tankk + tdrd4 + tdat + thtt + tcomt;

phgentot = parhostc * genetot ;

phtankkd = parhostc * tankkd ;

phtdrd4d = parhostc * tdrd4d ;

phtdatd = parhostc * tdatd ;

phthttd = parhostc * thttd ;

phtcomtd = parhostc * tcomtd ;

phtankkr = parhostc * tankkr ;

phtdrd4r = parhostc * tdrd4r ;

phtdatr = parhostc * tdatr ;

phthttr = parhostc * thttr ;

phtcomtr = parhostc * tcomtr ;

ANALYSIS: iterations = 10000;

MODEL: f14host on female (B01)

parhostc (B02)

genetot (B03)

phgentot (B04);

OUTPUT: SAMPSTAT MODINDICES(0) TECH1 TECH4 standardized residual;

**Model 3**

TITLE: SEM Model 3, using Total Gene index

sample size = 281

E main effect = 0 = parent HOSTILITY to target aged 15

G main effect = sum across 5 SNPs

GxE interact = estimated

outcome = target HOSTILITY to partner aged 31

DATA: FILE IS april352.dat;

VARIABLE: NAMES ARE

famid

female male

parwarm parhost tarwarm tarhost f14warm f14host

tankk tdrd4 tdat thtt tcomt

tankkd tdrd4d tdatd thttd tcomtd

tankkr tdrd4r tdatr thttr tcomtr

complet;

USEVARIABLES are

f14host female parhostc genetot phgentot;

MISSING are .;

USEOBSERVATIONS = complet eq 1;

DEFINE: parwarmc = parwarm - 4.4937722;

parhostc = parhost - 3.6304840;

genetot = tankk + tdrd4 + tdat + thtt + tcomt;

phgentot = parhostc * genetot ;

phtankkd = parhostc * tankkd ;

phtdrd4d = parhostc * tdrd4d ;

phtdatd = parhostc * tdatd ;

phthttd = parhostc * thttd ;

phtcomtd = parhostc * tcomtd ;

phtankkr = parhostc * tankkr ;

phtdrd4r = parhostc * tdrd4r ;

phtdatr = parhostc * tdatr ;

phthttr = parhostc * thttr ;

phtcomtr = parhostc * tcomtr ;

ANALYSIS: iterations = 10000;

MODEL: f14host on female (B01)

parhostc@0 (B02)

genetot (B03)

phgentot (B04);

OUTPUT: SAMPSTAT MODINDICES(0) TECH1 TECH4 standardized residual;

**Model 4**

TITLE: SEM Model 4, using Total Gene index

sample size = 281

E main effect = 0 = parent HOSTILITY to target aged 15

G main effect = 0 = sum across 5 SNPs

GxE interact = estimated

outcome = target HOSTILITY to partner aged 31

DATA: FILE IS april352.dat;

VARIABLE: NAMES ARE

famid

female male

parwarm parhost tarwarm tarhost f14warm f14host

tankk tdrd4 tdat thtt tcomt

tankkd tdrd4d tdatd thttd tcomtd

tankkr tdrd4r tdatr thttr tcomtr

complet;

USEVARIABLES are

f14host female parhostc genetot phgentot;

MISSING are .;

USEOBSERVATIONS = complet eq 1;

DEFINE: parwarmc = parwarm - 4.4937722;

parhostc = parhost - 3.6304840;

genetot = tankk + tdrd4 + tdat + thtt + tcomt;

phgentot = parhostc * genetot ;

phtankkd = parhostc * tankkd ;

phtdrd4d = parhostc * tdrd4d ;

phtdatd = parhostc * tdatd ;

phthttd = parhostc * thttd ;

phtcomtd = parhostc * tcomtd ;

phtankkr = parhostc * tankkr ;

phtdrd4r = parhostc * tdrd4r ;

phtdatr = parhostc * tdatr ;

phthttr = parhostc * thttr ;

phtcomtr = parhostc * tcomtr ;

ANALYSIS: iterations = 10000;

MODEL: f14host on female (B01)

parhostc@0 (B02)

genetot @0 (B03)

phgentot (B04);

OUTPUT: SAMPSTAT MODINDICES(0) TECH1 TECH4 standardized residual;

**Model 5**

TITLE: SEM Model 5, using 10 Separate SNP scores

sample size = 281

E main effect = parent HOSTILITY

G main effect = 10 separate SNPs

GxE interact = not included

outcome = target HOSTILITY to partner aged 31

DATA: FILE IS april352.dat;

VARIABLE: NAMES ARE

famid

female male

parwarm parhost tarwarm tarhost f14warm f14host

tankk tdrd4 tdat thtt tcomt

tankkd tdrd4d tdatd thttd tcomtd

tankkr tdrd4r tdatr thttr tcomtr

complet;

USEVARIABLES are

f14host female

tankkd tdrd4d tdatd thttd tcomtd

tankkr tdrd4r tdatr thttr tcomtr

parhostc;

MISSING are .;

USEOBSERVATIONS = complet eq 1;

DEFINE: parwarmc = parwarm - 4.4937722;

parhostc = parhost - 3.6304840;

genetot = tankk + tdrd4 + tdat + thtt + tcomt;

phgentot = parhostc * genetot ;

phtankkd = parhostc * tankkd ;

phtdrd4d = parhostc * tdrd4d ;

phtdatd = parhostc * tdatd ;

phthttd = parhostc * thttd ;

phtcomtd = parhostc * tcomtd ;

phtankkr = parhostc * tankkr ;

phtdrd4r = parhostc * tdrd4r ;

phtdatr = parhostc * tdatr ;

phthttr = parhostc * thttr ;

phtcomtr = parhostc * tcomtr ;

ANALYSIS: iterations = 10000;

MODEL: f14host on female (B01)

parhostc (B02)

tankkd (B03)

tdrd4d (B04)

tdatd (B05)

thttd (B06)

tcomtd (B07)

tankkr (B08)

tdrd4r (B09)

tdatr (B10)

thttr (B11)

tcomtr (B12);

OUTPUT: SAMPSTAT MODINDICES(0) TECH1 TECH4 standardized residual;

**Model 6**

TITLE: SEM Model 6, using 10 Separate SNP scores

sample size = 281

E main effect = parent HOSTILITY

G main effect = 10 separate SNPs

GxE interact = estimated

outcome = target HOSTILITY to partner aged 31

DATA: FILE IS april352.dat;

VARIABLE: NAMES ARE

famid

female male

parwarm parhost tarwarm tarhost f14warm f14host

tankk tdrd4 tdat thtt tcomt

tankkd tdrd4d tdatd thttd tcomtd

tankkr tdrd4r tdatr thttr tcomtr

complet;

USEVARIABLES are

f14host female

tankkd tdrd4d tdatd thttd tcomtd

tankkr tdrd4r tdatr thttr tcomtr

parhostc

phtankkd phtdrd4d phtdatd phthttd phtcomtd

phtankkr phtdrd4r phtdatr phthttr phtcomtr

;

MISSING are .;

USEOBSERVATIONS = complet eq 1;

DEFINE: parwarmc = parwarm - 4.4937722;

parhostc = parhost - 3.6304840;

genetot = tankk + tdrd4 + tdat + thtt + tcomt;

phgentot = parhostc * genetot ;

phtankkd = parhostc * tankkd ;

phtdrd4d = parhostc * tdrd4d ;

phtdatd = parhostc * tdatd ;

phthttd = parhostc * thttd ;

phtcomtd = parhostc * tcomtd ;

phtankkr = parhostc * tankkr ;

phtdrd4r = parhostc * tdrd4r ;

phtdatr = parhostc * tdatr ;

phthttr = parhostc * thttr ;

phtcomtr = parhostc * tcomtr ;

ANALYSIS: iterations = 10000;

MODEL: f14host on female (B01)

parhostc (B02)

tankkd (B03)

tdrd4d (B04)

tdatd (B05)

thttd (B06)

tcomtd (B07)

tankkr (B08)

tdrd4r (B09)

tdatr (B10)

thttr (B11)

tcomtr (B12)

phtankkd (B13)

phtdrd4d (B14)

phtdatd (B15)

phthttd (B16)

phtcomtd (B17)

phtankkr (B18)

phtdrd4r (B19)

phtdatr (B20)

phthttr (B21)

phtcomtr (B22)

;

OUTPUT: SAMPSTAT MODINDICES(0) TECH1 TECH4 standardized residual;

**Model 7**

TITLE: SEM Model 7, using 10 Separate SNP scores

sample size = 281

E main effect = parent HOSTILITY = FIXED at 0

G main effect = constrained equal

GxE interact = constrained equal

outcome = target HOSTILITY to partner aged 31

DATA: FILE IS april352.dat;

VARIABLE: NAMES ARE

famid

female male

parwarm parhost tarwarm tarhost f14warm f14host

tankk tdrd4 tdat thtt tcomt

tankkd tdrd4d tdatd thttd tcomtd

tankkr tdrd4r tdatr thttr tcomtr

complet;

USEVARIABLES are

f14host female

tankkd tdrd4d tdatd thttd tcomtd

tankkr tdrd4r tdatr thttr tcomtr

parhostc

phtankkd phtdrd4d phtdatd phthttd phtcomtd

phtankkr phtdrd4r phtdatr phthttr phtcomtr

;

MISSING are .;

USEOBSERVATIONS = complet eq 1;

DEFINE: parwarmc = parwarm - 4.4937722;

parhostc = parhost - 3.6304840;

genetot = tankk + tdrd4 + tdat + thtt + tcomt;

phgentot = parhostc * genetot ;

phtankkd = parhostc * tankkd ;

phtdrd4d = parhostc * tdrd4d ;

phtdatd = parhostc * tdatd ;

phthttd = parhostc * thttd ;

phtcomtd = parhostc * tcomtd ;

phtankkr = parhostc * tankkr ;

phtdrd4r = parhostc * tdrd4r ;

phtdatr = parhostc * tdatr ;

phthttr = parhostc * thttr ;

phtcomtr = parhostc * tcomtr ;

ANALYSIS: iterations = 10000;

MODEL: f14host on female (B01)

parhostc@0 (B02)

tankkd (B03)

tdrd4d (B03)

tdatd (B03)

thttd (B03)

tcomtd (B03)

tankkr (B03)

tdrd4r (B03)

tdatr (B03)

thttr (B03)

tcomtr (B03)

phtankkd (B13)

phtdrd4d (B13)

phtdatd (B13)

phthttd (B13)

phtcomtd (B13)

phtankkr (B13)

phtdrd4r (B13)

phtdatr (B13)

phthttr (B13)

phtcomtr (B13)

;

OUTPUT: SAMPSTAT MODINDICES(0) TECH1 TECH4 standardized residual;

**Model 8**

TITLE: SEM Model 8, using 10 Separate SNP scores

sample size = 281

E main effect = parent HOSTILITY = FIXED at 0

G main effect = 10 separate SNPS = FIXED at 0

GxE interact = constrained equal

outcome = target HOSTILITY to partner aged 31

DATA: FILE IS april352.dat;

VARIABLE: NAMES ARE

famid

female male

parwarm parhost tarwarm tarhost f14warm f14host

tankk tdrd4 tdat thtt tcomt

tankkd tdrd4d tdatd thttd tcomtd

tankkr tdrd4r tdatr thttr tcomtr

complet;

USEVARIABLES are

f14host female

tankkd tdrd4d tdatd thttd tcomtd

tankkr tdrd4r tdatr thttr tcomtr

parhostc

phtankkd phtdrd4d phtdatd phthttd phtcomtd

phtankkr phtdrd4r phtdatr phthttr phtcomtr

;

MISSING are .;

USEOBSERVATIONS = complet eq 1;

DEFINE: parwarmc = parwarm - 4.4937722;

parhostc = parhost - 3.6304840;

genetot = tankk + tdrd4 + tdat + thtt + tcomt;

phgentot = parhostc * genetot ;

phtankkd = parhostc * tankkd ;

phtdrd4d = parhostc * tdrd4d ;

phtdatd = parhostc * tdatd ;

phthttd = parhostc * thttd ;

phtcomtd = parhostc * tcomtd ;

phtankkr = parhostc * tankkr ;

phtdrd4r = parhostc * tdrd4r ;

phtdatr = parhostc * tdatr ;

phthttr = parhostc * thttr ;

phtcomtr = parhostc * tcomtr ;

ANALYSIS: iterations = 10000;

MODEL: f14host on female (B01)

parhostc@0 (B02)

tankkd @0 (B03)

tdrd4d @0 (B03)

tdatd @0 (B03)

thttd @0 (B03)

tcomtd @0 (B03)

tankkr @0 (B03)

tdrd4r @0 (B03)

tdatr @0 (B03)

thttr @0 (B03)

tcomtr @0 (B03)

phtankkd (B13)

phtdrd4d (B13)

phtdatd (B13)

phthttd (B13)

phtcomtd (B13)

phtankkr (B13)

phtdrd4r (B13)

phtdatr (B13)

phthttr (B13)

phtcomtr (B13)

;

OUTPUT: SAMPSTAT MODINDICES(0) TECH1 TECH4 standardized residual;

**lavaan script for analyses of Masarik et al. (2014) data**

library(lavaan)

library(psych)

#------------------------------------------------------------------------------------#

# reading data set into R (data in ASCII format, with . for missing values) #

# change element in na.strings statement to substitute a different code #

# for missing values (e.g., -99) #

#------------------------------------------------------------------------------------#

setwd('C:/Users/…/data/april/lavaan')

april352 = read.table(file = 'april352.dat', na = '.')

dim(april352)

names(april352) =

c('famid' , 'female' , 'male' , 'parpeng' , 'parhost' ,

'tarpeng' , 'tarhost' , 'f14peng' , 'f14host' ,

'tankk' , 'tdrd4' , 'tdat' , 'thtt' , 'tcomt' ,

'tankkd' , 'tdrd4d' , 'tdatd' , 'thttd' , 'tcomtd' ,

'tankkr' , 'tdrd4r' , 'tdatr' , 'thttr' , 'tcomtr' ,

'complet' )

head(april352)

#------------------------------------------------------------------------------------#

# subset april352 (N = 352) to complete data april281 (N = 281) #

#------------------------------------------------------------------------------------#

april281 = april352[ which(april352$complet == 1) , ]

dim(april281)

#------------------------------------------------------------------------------------#

#------------------------------------------------------------------------------------#

# program to analyze GxE interactions for HOSTILITY at WAVE 14 #

# AND #

# using the SEM fitting function in lavaan #

#------------------------------------------------------------------------------------#

# recode variables, create product vectors, etc. #

#------------------------------------------------------------------------------------#

# dealing first with positive engagement recodes and product vectors #

#------------------------------------------------------------------------------------#

april281$parpengc = april281$parpeng - 4.4937722;

april281$genetot = april281$tankk + april281$tdrd4 + april281$tdat + april281$thtt +

april281$tcomt;

april281$pegentot = april281$parpengc * april281$genetot ;

april281$petankkd = april281$parpengc * april281$tankkd ;

april281$petdrd4d = april281$parpengc * april281$tdrd4d ;

april281$petdatd = april281$parpengc * april281$tdatd ;

april281$pethttd = april281$parpengc * april281$thttd ;

april281$petcomtd = april281$parpengc * april281$tcomtd ;

april281$petankkr = april281$parpengc * april281$tankkr ;

april281$petdrd4r = april281$parpengc * april281$tdrd4r ;

april281$petdatr = april281$parpengc * april281$tdatr ;

april281$pethttr = april281$parpengc * april281$thttr ;

april281$petcomtr = april281$parpengc * april281$tcomtr ;

#------------------------------------------------------------------------------------#

# now dealing with hostility recodes and product vectors #

#------------------------------------------------------------------------------------#

april281$parhostc = april281$parhost - 3.6304840;

april281$phgentot = april281$parhostc * april281$genetot ;

april281$phtankkd = april281$parhostc * april281$tankkd ;

april281$phtdrd4d = april281$parhostc * april281$tdrd4d ;

april281$phtdatd = april281$parhostc * april281$tdatd ;

april281$phthttd = april281$parhostc * april281$thttd ;

april281$phtcomtd = april281$parhostc * april281$tcomtd ;

april281$phtankkr = april281$parhostc * april281$tankkr ;

april281$phtdrd4r = april281$parhostc * april281$tdrd4r ;

april281$phtdatr = april281$parhostc * april281$tdatr ;

april281$phthttr = april281$parhostc * april281$thttr ;

april281$phtcomtr = april281$parhostc * april281$tcomtr ;

#------------------------------------------------------------------------------------#

# Model 1: Predict Target Hostility: from E and G main effects #

#------------------------------------------------------------------------------------#

model_01 = '

f14host ~ female + parhostc + genetot

f14host ~ 1

'

fit_01 = sem(model_01, data = april281)

summary(fit_01, fit.measures = TRUE, standardized = TRUE, modindices = TRUE)

inspect(fit_01)

#------------------------------------------------------------------------------------#

# Model 1a: Alternative model to Predict Target Hostility: from E and G main effects #

#------------------------------------------------------------------------------------#

model_01a = '

f14host ~ female + parhostc + genetot + 0*phgentot

f14host ~ 1

'

fit_01a = sem(model_01a, data = april281)

summary(fit_01a, fit.measures = TRUE, standardized = TRUE, modindices = TRUE)

inspect(fit_01a)

#------------------------------------------------------------------------------------#

# Model 2: Predict Target Hostility: from E and G main effects, add GxE #

#------------------------------------------------------------------------------------#

model_02 = '

f14host ~ female + parhostc + genetot + phgentot

f14host ~ 1

'

fit_02 = sem(model_02, data = april281)

summary(fit_02, fit.measures = TRUE, standardized = TRUE, modindices = TRUE)

inspect(fit_02)

#------------------------------------------------------------------------------------#

# Model 3: Predict Target Hostility: from G and GxE, fix E = 0 #

#------------------------------------------------------------------------------------#

model_03 = '

f14host ~ female + 0*parhostc + genetot + phgentot

f14host ~ 1

'

fit_03 = sem(model_03, data = april281)

summary(fit_03, fit.measures = TRUE, standardized = TRUE, modindices = TRUE)

inspect(fit_03)

#------------------------------------------------------------------------------------#

# Model 4: Predict Target Hostility: from GxE, fix E = 0 and G = 0 #

#------------------------------------------------------------------------------------#

model_04 = '

f14host ~ female + 0*parhostc + 0*genetot + phgentot

f14host ~ 1

'

fit_04 = sem(model_04, data = april281)

summary(fit_04, fit.measures = TRUE, standardized = TRUE, modindices = TRUE)

inspect(fit_04)

#------------------------------------------------------------------------------------#

# Model 5: Predict Target Hostility: from E and 10 G SNPs #

#------------------------------------------------------------------------------------#

model_05 = '

f14host ~ female + parhostc +

B01*tankkd + B02*tdrd4d + B03*tdatd + B04*thttd + B05*tcomtd +

B06*tankkr + B07*tdrd4r + B08*tdatr + B09*thttr + B10*tcomtr

f14host ~ 1

'

fit_05 = sem(model_05, data = april281)

summary(fit_05, fit.measures = TRUE, standardized = TRUE, modindices = TRUE)

inspect(fit_05)

#------------------------------------------------------------------------------------#

# Model 6: Predict Target Hostility: from E, 10 G SNPs, add 10 GxE components #

#------------------------------------------------------------------------------------#

model_06 = '

f14host ~ female + parhostc +

B01*tankkd + B02*tdrd4d + B03*tdatd + B04*thttd + B05*tcomtd +

B06*tankkr + B07*tdrd4r + B08*tdatr + B09*thttr + B10*tcomtr +

B11*phtankkd + B12*phtdrd4d + B13*phtdatd + B14*phthttd + B15*phtcomtd +

B16*phtankkr + B17*phtdrd4r + B18*phtdatr + B19*phthttr + B20*phtcomtr

f14host ~ 1

'

fit_06 = sem(model_06, data = april281)

summary(fit_06, fit.measures = TRUE, standardized = TRUE, modindices = TRUE)

inspect(fit_06)

#------------------------------------------------------------------------------------#

# Model 7: Predict Target Hostility: E = 0, 10 G SNPs =, 10 GxE components = #

#------------------------------------------------------------------------------------#

model_07 = '

f14host ~ female + 0*parhostc +

B01*tankkd + B01*tdrd4d + B01*tdatd + B01*thttd + B01*tcomtd +

B01*tankkr + B01*tdrd4r + B01*tdatr + B01*thttr + B01*tcomtr +

B11*phtankkd + B11*phtdrd4d + B11*phtdatd + B11*phthttd + B11*phtcomtd +

B11*phtankkr + B11*phtdrd4r + B11*phtdatr + B11*phthttr + B11*phtcomtr

f14host ~ 1

'

fit_07 = sem(model_07, data = april281)

summary(fit_07, fit.measures = TRUE, standardized = TRUE)

inspect(fit_07)

lavTestScore(fit_07, uni = TRUE)

#------------------------------------------------------------------------------------#

# Model 8: Predict Target Hostility: E = 0, 10 G SNPs = 0, 10 GxE components = #

#------------------------------------------------------------------------------------#

model_08 = '

f14host ~ female + 0*parhostc +

0*tankkd + 0*tdrd4d + 0*tdatd + 0*thttd + 0*tcomtd +

0*tankkr + 0*tdrd4r + 0*tdatr + 0*thttr + 0*tcomtr +

B02*phtankkd + B02*phtdrd4d + B02*phtdatd + B02*phthttd + B02*phtcomtd +

B02*phtankkr + B02*phtdrd4r + B02*phtdatr + B02*phthttr + B02*phtcomtr

f14host ~ 1

'

fit_08 = sem(model_08, data = april281)

summary(fit_08, fit.measures = TRUE, standardized = TRUE)

inspect(fit_08)

lavTestScore(fit_08, uni = TRUE)
